# Expression profiling and regulatory network of cucumber microRNAs and their putative target genes in response to cucumber green mottle mosaic virus infection

**DOI:** 10.1007/s00705-019-04152-w

**Published:** 2019-02-24

**Authors:** Chaoqiong Liang, Huawei Liu, Jianjun Hao, Jianqiang Li, Laixin Luo

**Affiliations:** 1 Department of Plant Pathology, China Agricultural University, Beijing, 100193 People’s Republic of China; 2 Beijing Key Laboratory of Seed Disease Testing and Control, China Agricultural University, Beijing, 100193 People’s Republic of China; 3 College of Horticulture, Nanjing Agricultural University, Nanjing, 210095 People’s Republic of China; 4 School of Food and Agriculture, The University of Maine, Orono, ME 04469 USA

## Abstract

Cucumber green mottle mosaic virus (CGMMV) is an important pathogen of cucumber (*Cucumis sativus*). The molecular mechanisms mediating host-pathogen interactions are likely to be strongly influenced by microRNAs (miRNAs), which are known to regulate gene expression during the disease cycle. This study focused on 14 miRNAs (miR159, miR169, miR172, miR838, miR854, miR5658, csa-miRn1-3p, csa-miRn2-3p, csa-miRn3-3p, csa-miRn4-5p, csa-miRn5-5p, csa-miRn6-3p, csa-miRn7-5p and csa-miRn8-3p) and their target genes. The data collected was used to construct a regulatory network of miRNAs and target genes associated with cucumber-CGMMV interactions, which identified 608 potential target genes associated with all of the miRNAs except csa-miRn7-5p. Five of the miRNAs (miR159, miR838, miR854, miR5658 and csa-miRn6-3p) were found to be mutually linked by target genes, while another eight (miR169, miR172, csa-miRn1-3p, csa-miRn2-3p, csa-miRn3-3p, csa-miRn4-5p, csa-miRn5-5p and csa-miRn8-3p) formed subnetworks that did not display any connectivity with other miRNAs or their target genes. Reverse transcription quantitative real-time PCR (RT-qPCR) was used to analyze the expression levels of the different miRNAs and their putative target genes in leaf, stem and root samples of cucumber over a 42-day period after inoculation with CGMMV. A positive correlation was found between some of the miRNAs and their respective target genes, although for most, the response varied greatly depending on the time point, indicating that additional factors are likely to be involved in the interaction between cucumber miRNAs and their target genes. Several miRNAs, including miR159 and csa-miRn6-3p, were linked to target genes that have been associated with plant responses to disease. A model linking miRNAs, their targets and downstream biological processes is proposed to indicate the roles of these miRNAs in the cucumber-CGMMV pathosystem.

**Electronic supplementary material:**

The online version of this article (10.1007/s00705-019-04152-w) contains supplementary material, which is available to authorized users.

## Introduction

Cucumber (*Cucumis sativus*) is a widely cultivated crop with an annual production of approximately 75 million tons worldwide (http://faostat3.fao.org). Viruses, including cucumber green mottle mosaic virus (CGMMV), are one of the main constraints on cucumber production. CGMMV is a member of the genus *Tobamovirus* and is readily transmitted by mechanical damage, infected soil, seeds and propagation stocks [1–7]. CGMMV has been reported in many countries including the UK, Pakistan, Indian, Israel, Japan, China, Greece and the USA [8–16]. The virus has a narrow host range and is confined to members of the family Cucurbitaceae, which includes cucumber, squash (*Cucurbita moschata*) and watermelon (*Citrullus lanatus*) [10, 17, 18]. Cucumbers infected with CGMMV exhibit characteristic mosaic symptoms in their leaves, with severe infections causing stunted growth and the production of distorted fruits [19, 20].

There are no effective methods to control diseases caused by CGMMV, and there are no resistant cucumber varieties. Consequently, disease control depends primarily on the management and certification of seeds and seedlings to avoid the introduction of the virus to areas of cucumber production. However, recent research has shown that seed disinfection treatments are insufficient to completely eliminate CGMMV contamination [21]. Fortunately, there is increasing evidence that molecular breeding could be a potential means of screening disease-resistant varieties and that microRNA (miRNA)-mediated RNA silencing could be used to control viral diseases [22, 23]. In this case, the negative regulation of expression by miRNAs that target viral suppressors of RNA silencing (VSRs) has been exploited to induce viral resistance in plants [22, 23].

MicroRNAs are a large group of small (21 to 24 nucleotides) non-coding RNA molecules found in eukaryotic cells [24]. Previous research has shown that miRNAs play important roles in posttranscriptional regulation of the expression of many genes, particularly those involved in cell differentiation, proliferation and apoptosis, via silencing of their mRNAs. Many miRNAs from a wide range of species have been characterized in detail, defining their sequence, structure and function. However, many miRNAs are known to be species-specific or have specific functions depending on the plant species. These miRNAs have diverse sequences and can repress the expression of many different target genes [25, 26].

The miRNAs of plants are known to be involved in an array of developmental processes and stress responses [27], and their expression profiles can be significantly modified during infection by plant pathogens [28]. Indeed, there is a growing body of evidence documenting the role of miRNAs in plant-pathogen interactions and their effect on the regulation of transcriptional or post-transcriptional processes [29]. For example, in *Arabidopsis*, at least three miRNAs (miR163, miR164 and miR167) are known to accumulate in response to tobacco mosaic virus (TMV) infection [30]. MiR6019 and miR6020 have been found to confer resistance to TMV in *Nicotiana tabacum* via nucleotide-binding (NB) leucine-rich repeat (LRR) immune receptor gene regulation [31]. Lang et al. [32] described the dynamic expression of 72 miRNAs in tomato plants inoculated with cucumber mosaic virus, predicted putative target genes. and discussed correlations between the accumulation of the miRNAs and pathogenesis. Xu et al. [33] investigated rice miRNAs responding to southern rice black-streaked dwarf virus (SRBSDV) infection and found that the expression levels of 56 miRNAs were altered in the diseased plants and that the miR164, miR396, miR530, miR1846 and miR1858 families in particular were associated with the development of symptoms. There have also been reports regarding the role of miRNAs in diseased cucumber. For example, seven novel candidate miRNAs have been identified in cucumber that respond to infection with hop stunt viroid [34], and a comprehensive parallel transcriptome and small RNAome sequencing analysis was conducted by Burkhardt and Day [29], identifying changes in gene and miRNA expression associated with the resistant/susceptible interactions of cucumber and *Pseudoperonospora cubensis*. Specifically, 28 conserved miRNA families target transcription factors, including miR156, miR157, miR169, and miR171, which target squamosa promoter-binding transcription factors, miR159 and miR319, which target MYB transcription factors, and miR166, which targets class III homeobox leucine zipper proteins [29]. The study also identified a novel cucumber-specific miR482 predicted to target a coiled-coil, a nucleotide binding-site, a leucine-rich repeat (CC-NBS-LRR), the *R* gene, and the mildew resistance locus O (MLO)-like protein, which plays a role in mediating defense against powdery mildew [29]. A role of miRNAs has also been suggested in plant-pathogen interactions associated with CGMMV infection. For example, Sun et al. [35] found that six miRNAs were upregulated in watermelon in response to CGMMV infection, which targeted genes involved in a diverse range of biological processes, including plant cell wall morphogenesis, plant hormone signaling, intracellular transport, primary and secondary metabolism, induction of defense-related proteins, and regulation of virus replication. Furthermore, a study conducted in our own laboratory identified 23 known miRNAs and eight novel ones from the leaf tissues of CGMMV-infected cucumber plants [36]. In this case, many of the predicted target genes were found to be involved in metabolic processes (166 pathways) and genetic information processes (40 pathways), and to a lesser degree the biosynthesis of secondary metabolites (12 pathways) [36]. The study also confirmed the expression of three of the novel miRNAs and three of the putative candidate miRNAs, and identified an additional 82 conserved miRNAs by microarray profiling of CGMMV-infected cucumbers [36]. However, although the study provided evidence that CGMMV infection affects miRNA abundance, the expression profile and regulatory network of the cucumber miRNAs and their target genes were not investigated in detail.

The objectives of the current study were (1) to select and analyze miRNAs and putative target genes associated with the disease response of cucumber, (2) to analyze the expression patterns of the miRNAs and their putative target genes during infection with CGMMV, (3) to analyze the relationship between the expression levels of miRNAs with altered expression in response to CGMMV and their putative target genes, and (4) to explore the relationship between miRNA regulation and disease development in cucumber infected with CGMMV.

## Materials and methods

### Virus, seedlings, and inoculation

The CGMMV isolate used in the current study was collected from the Zhejiang Province of China, and its identity was confirmed using double antibody sandwich enzyme-linked immunosorbent assay (DAS-ELISA) (ELISA reagent set for CGMMV, SRA 45702/0096, Agdia, Elkhart, IN, USA). The virus was maintained and propagated *in vivo* on a cucumber host in accordance with the protocol of a previous study [7]. The cucumber seeds (cv. ‘Zhongnong 16’) used in the study were confirmed to be CGMMV-free by reverse transcription polymerase chain reaction (RT-PCR) and planted in an insect-proof greenhouse. Cucumber seedlings were artificially inoculated with CGMMV at the three-true-leaf stage, and seedlings treated with PBS-T buffer (phosphate-buffered saline containing 0.1% Tween 20, pH 7.4) were used as a negative control. Leaf, stem and root samples of the seedlings were collected at 1, 7, 14, 21, 28, 35 and 42 days post-inoculation (dpi) and stored in a -80 °C freezer for later use.

### RNA extraction and cDNA synthesis

miRNAs were extracted from leaf, stem and root samples using a miRcute miRNA Isolation Kit (DP501, Tiangen Biotech, Beijing, China) according to the protocol of the manufacturer. The cDNA was synthesized using a poly(A)-tailing method and a miRcute miRNA First-Strand cDNA Synthesis Kit (KR201-02, Tiangen Biotech, China) according to the manufacturer’s instructions and stored at -20 °C until required.

For target genes, total RNA from leaf, stem and root samples was extracted using TRIzol Reagent (Ambion, USA) according to the protocol of the manufacturer, and its quantity and quality were assessed using a NanoDrop 2000spectrophotometer (Thermo Scientific). RNA samples exhibiting an A_260_/A_280_ ratio of 1.8-2.0 and an A_260_/A_230_ ratio of 2.0-2.2 were selected for subsequent analysis. The cDNA was synthesized using a PrimeScript RT Reagent kit with gDNA Eraser (Perfect Real Time) (RR047A, TaKaRa, Kusatsu, Shiga, Japan) and stored at -20 °C until required.

### Selection of cucumber miRNAs in response to CGMMV infection

In 2015, cucumber miRNAs that are differentially regulated in response to CGMMV infection were identified by high-throughput sequencing. The microarray analysis of 88 miRNAs showed that the expression levels of miR159, miR169, miR172, miR838, miR854, miR5658, csa-miRn6-3p, csa-miRn7-5p and csa-miRn8-3p were significantly altered in CGMMV-infected cucumber leaves at 10, 30 and 50 dpi [36]. In this study, therefore, 14 miRNAs (miR159, miR169, miR172, miR838, miR854, miR5658, csa-miRn1-3p, csa-miRn2-3p, csa-miRn3-3p, csa-miRn4-5p, csa-miRn5-5p, csa-miRn6-3p, csa-miRn7-5p and csa-miRn8-3p) were selected from the chip expression profile results and further analyzed for their expression characteristics and role in regulatory networks.

### Prediction and analysis of target genes and construction of a regulatory network

Target genes of 14 miRNAs were predicted using the psRNATarget online tool (http://plantgrn.noble.org/psRNATarget/) [37]. Targets with scores < 4.0 were considered potential target genes. In plants, mismatches occurring around the center of the miRNA/mRNA complementary region tend to disable the cleavage activity of the miRNA-directed RNA-induced silencing complex (RISC); however, the binding of mRNA to the RISC still can block gene expression at the translational level [38]. The psRNATarget server reports translational inhibition potential when a mismatch is detected in the central complementary region of the small RNA sequence [37, 39].

The functions of various target genes were then assigned using Gene Ontology (GO) and Kyoto Encyclopedia of Genes and Genomes (KEGG) analysis in conjunction with Uni-protKB and the National Center for Biotechnology Information (NCBI) database. A regulatory network linking the miRNAs and their putative target genes was then constructed using Cytoscape 3.2.0 [40–42].

### RT-qPCR analysis of miRNAs and their putative target genes

Reverse transcription quantitative real-time PCR (RT-qPCR) was used to investigate the expression levels of cucumber miRNAs and their putative target genes associated with plant defense responses. The primers used in the study (Tables S1 and S2) were designed using Primer Premier 5.0 software (Premier Biosoft International, Palo Alto, CA, USA) [43] and synthesized by Sangon Biotech (Shanghai, China). The cucumber elongation factor 1-alpha (*EF-1α*) and *Ubiquitin* genes were used as the internal controls to normalize the RT-qPCR data.

RT-qPCR of miRNAs was carried out using a miRcute miRNA qPCR Detection Kit (SYBR Green) (FP401, Tiangen Biotech, China). PCR was performed in 20-μL reaction mixtures containing 10 μL of 2 × miRcute miRNA Premix (with SYBR & ROX), 1 μL of cDNA, 0.4 μL of each of the forward and reverse primers (10 μM), and 8.2 μL of RNase-free water and processed using an Applied Biosystems 7500 Fast Real-Time PCR System (Life Technologies, USA) with the following program: 94 °C for 2 min, followed by 40 cycles of 94 °C for 20 s and 60 °C for 34 s. The melting curves were generated at 95 °C after the reaction had been terminated (Fig. S1).

The expression levels of six genes (Csa4M022940.1, Csa4M015840.1, Csa5M175970.1, Csa6M520360.1, Csa2M224260.1 and Csa1M470280.1) targeted by six miRNAs (miR159, miR169, miR172, miR838, miR854 and miR5658) and two genes (Csa7M425940.1 and Csa4M045040.1) targeted by csa-miRn6-3p were investigated using RT-qPCR and the SYBR Premix Dimer Eraser System (RR091A, Takara, Japan). The PCR was conducted using 20-μL qPCR reaction mixtures containing 10 μL of SYBR Premix Dimer Eraser (2 ×), 0.6 μL of each of the forward and reverse primers (10 μM), 2 μL of cDNA, 0.4 μL of ROX DyeII(50 ×), and 6.4 μL of RNase-free water, and the following program was used: 95 °C for 30 s, followed by 40 cycles of 95 °C for 5 s, 55 °C for 30 s and 72 °C for 34 s. The melting curves were generated at 95 °C after the reaction had been terminated (Fig. S1). The PCR amplification specificities of eight target genes and two reference genes were confirmed by amplification of a single band with the expected size, and no primer-dimer formation was detected in agarose gel electrophoresis (Fig. S2).

Three biological replicates were used for each miRNA or target gene. All of the reactions were performed in triplicate, and the data were normalized using the 2^-△△Ct^ method: △△Ct = (Ct_miRNAs/target genes_ - Ct_*EF-1α* and *Ubiquitin*_) _infected_ - (Ct_miRNAs/target genes_ - Ct_*EF-1α* and *Ubiquitin*_) _control_ [44].

## Results

### Selection of cucumber miRNAs in response to CGMMV infection

Alignment of the unmapped sequences with cucumber genomic DNA and filtering according to the screening criteria for miRNAs indicated that 12 new miRNAs were isolated. However, analysis of the secondary structure of their precursors and their location in the arm of the stem-loop structure indicated that only eight of the 12 were confirmed as novel miRNAs by bioinformatic analysis using RNAfold software [36]. They were named csa-miRn1-3p, csa-miRn2-3p, csa-miRn3-3p, csa-miRn4-5p, csa-miRn5-5p, csa-miRn6-3p, csa-miRn7-5p and csa-miRn8-3p. Microarray analysis of miRNAs showed that the expression levels of miR159, miR169, miR172, miR838, miR854, miR5658, csa-miRn6-3p, csa-miRn7-5p and csa-miRn8-3p were significantly altered in the CGMMV-infected cucumber leaves at 10, 30 and 50 dpi. Therefore, 14 miRNAs (miR159, miR169, miR172, miR838, miR854, miR5658, csa-miRn1-3p, csa-miRn2-3p, csa-miRn3-3p, csa-miRn4-5p, csa-miRn5-5p, csa-miRn6-3p, csa-miRn7-5p and csa-miRn8-3p were examined in this study.

### Prediction of target genes and construction of a regulatory network

**Fig. 1:**
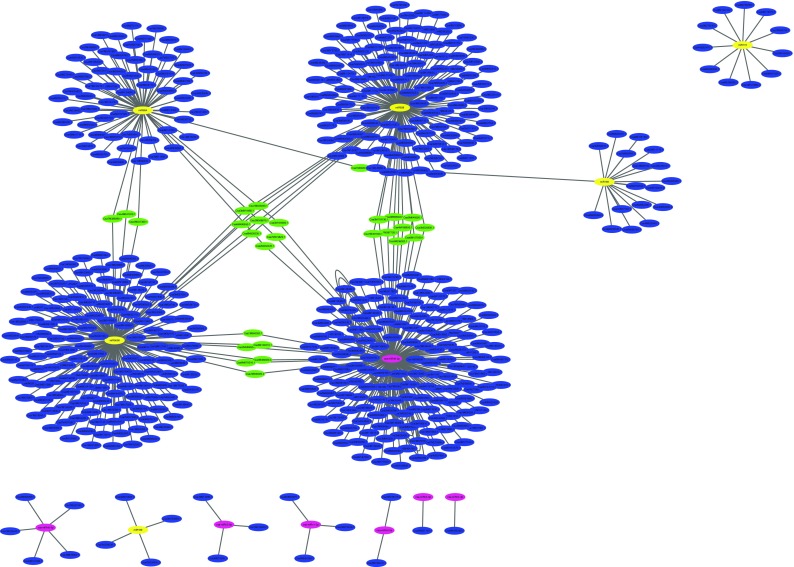
Regulatory network linking miRNAs to their putative target genes. Yellow nodes indicate known miRNAs, pink nodes indicate novel miRNAs, and blue nodes indicate the putative target genes. Green nodes indicate putative target genes that can be regulated by more than one miRNA. The lines represent connections between nodes that exhibit a high degree of correlation

psRNATarget analysis identified 608 target genes associated with the 13 cucumber miRNAs assessed: 397 associated with the six miRNAs (miR159, miR169, miR172, miR838, miR854 and miR5658) and 211 associated with seven miRNAs (csa-miRn1-3p, csa-miRn2-3p, csa-miRn3-3p, csa-miRn4-5p, csa-miRn5-5p, csa-miRn6-3p, csa-miRn8-3p) (Table S3), but none with the eighth, csa-miRn7-5p. However, the regulatory network produced indicated that individual target genes could be regulated by multiple miRNAs (Fig. 1). Five miRNAs (miR159, miR838, miR854, miR5658 and csa-miRn6-3p) formed a complex regulatory network linked by 27 target genes that were influenced by two or more miRNAs. This primary network encompassed the vast majority of the target genes, with only a few forming much smaller discrete subnetworks associated with miR169, miR172, csa-miRn1-3p, csa-miRn2-3p, csa-miRn3-3p, csa-miRn4-5p, csa-miRn5-5p and csa-miRn8-3p.

GO and KEGG analyses indicated that the majority of the target genes were associated with plant growth, metabolism and cell morphogenesis, and processes associated with responses to disease (Table S4). Sixteen of the target genes associated with plant defense in cucumber, including representatives regulated by the six known miRNAs and three of the novel miRNAs, were selected for further analysis (Table 1). Those associated with the known miRNAs miR159, miR169, miR172, miR838, miR854 and miR5658 were predicted to encode the following proteins: myb-related protein MYB29-like, putative disease resistance protein RGA3-like, ethylene-responsive transcription factor RAP2-7-like, MACPF domain-containing protein CAD1-like, probable WRKY transcription factor 21-like, and LRR receptor-like serine/threonine-protein kinase FLS2-like, respectively. Those associated with the novel miRNAs csa-miRn1-3p, csa-miRn5-5p and csa-miRn6-3p included UDP-glycosyltransferase 73B3-like, glucan endo-1,3-beta-glucosidase, pathogenesis-related homeodomain protein-like, vicilin-like antimicrobial peptides 2-1-like, ethylene-responsive transcription factor CRF4-like, putative disease resistance protein RGA4-like, linoleate 9S-lipoxygenase 1-like, RNA polymerase II C-terminal domain phosphatase-like 3-like, and probable dehydrin LEA-like.

**Table 1:** Putative functions of 16 target genes associated with nine miRNAs in cucumber

miRNA	Target accession	Inhibition type	Target	Putative function
miR159	Csa4M022940.1	Cleavage	Transcription factor MYB29-like	Abiotic stress tolerance and biotic stress resistance
miR169	Csa4M015840.1	Translation	Putative disease resistance protein RGA3-like	Disease resistance that triggers a defense system that restricts pathogen growth
miR172	Csa5M175970.1	Cleavage	Ethylene-responsive transcription factor RAP2-7-like	May be involved in the regulation of gene expression by stress factors and by components of stress signal transduction pathways
miR838	Csa6M520360.1	Cleavage	MACPF(membrane attack complex and perforin) domain-containing protein CAD1-like	CAD1 protein negatively controls the SA-mediated pathway of programmed cell death during plant immunity responses.
miR854	Csa2M224260.1	Cleavage	Probable WRKY transcription factor 21-like	Defense responses
miR5658	Csa1M470280.1	Cleavage	LRR receptor-like serine/threonine-protein kinase FLS2-like	Disease resistance
csa-miRn1-3p	Csa7M073450.1	Cleavage	UDP-glycosyltransferase 73B3-like	Pathogen-responsive gene
csa-miRn5-5p	Csa1M616240.1	Cleavage	Glucan endo-1,3-beta-glucosidase, basic isoform-like	Plant defense-related product targeting fungal pathogens
csa-miRn6-3p	Csa1M424880.1	Cleavage	Pathogenesis-related homeodomain protein-like	Defense response
	Csa3M146460.1	Cleavage	Vicilin-like antimicrobial peptides 2-1-like	Defense response to bacteria and fungi resulting in the death of the foreign organism
	Csa5M139630.1	Cleavage	Ethylene-responsive transcription factor CRF4-like	May be involved in the regulation of gene expression by stress factors and by components of stress signal transduction pathways
	Csa7M425940.1	Cleavage	Putative disease resistance protein RGA4-like	Disease resistance that triggers a defense system that restricts pathogen growth
	Csa7M374620.1	Cleavage	Linoleate 9S-lipoxygenase 1-like	Influence on plant physiology including growth and development, pest resistance, and senescence or responses to wounding.
	Csa6M091910.1	Cleavage	RNA polymerase II C-terminal domain phosphatase-like 3-like	Modulates plant growth, stress (cold), and phytohormones responses
	Csa4M045040.1	Cleavage	Probable dehydrin LEA-like	Response to stress and water
	Csa6M133770.1	Cleavage	Ethylene-responsive transcription factor CRF4-like	May be involved in the regulation of gene expression by stress factors and by components of stress signal transduction pathways

**Fig. 2:**
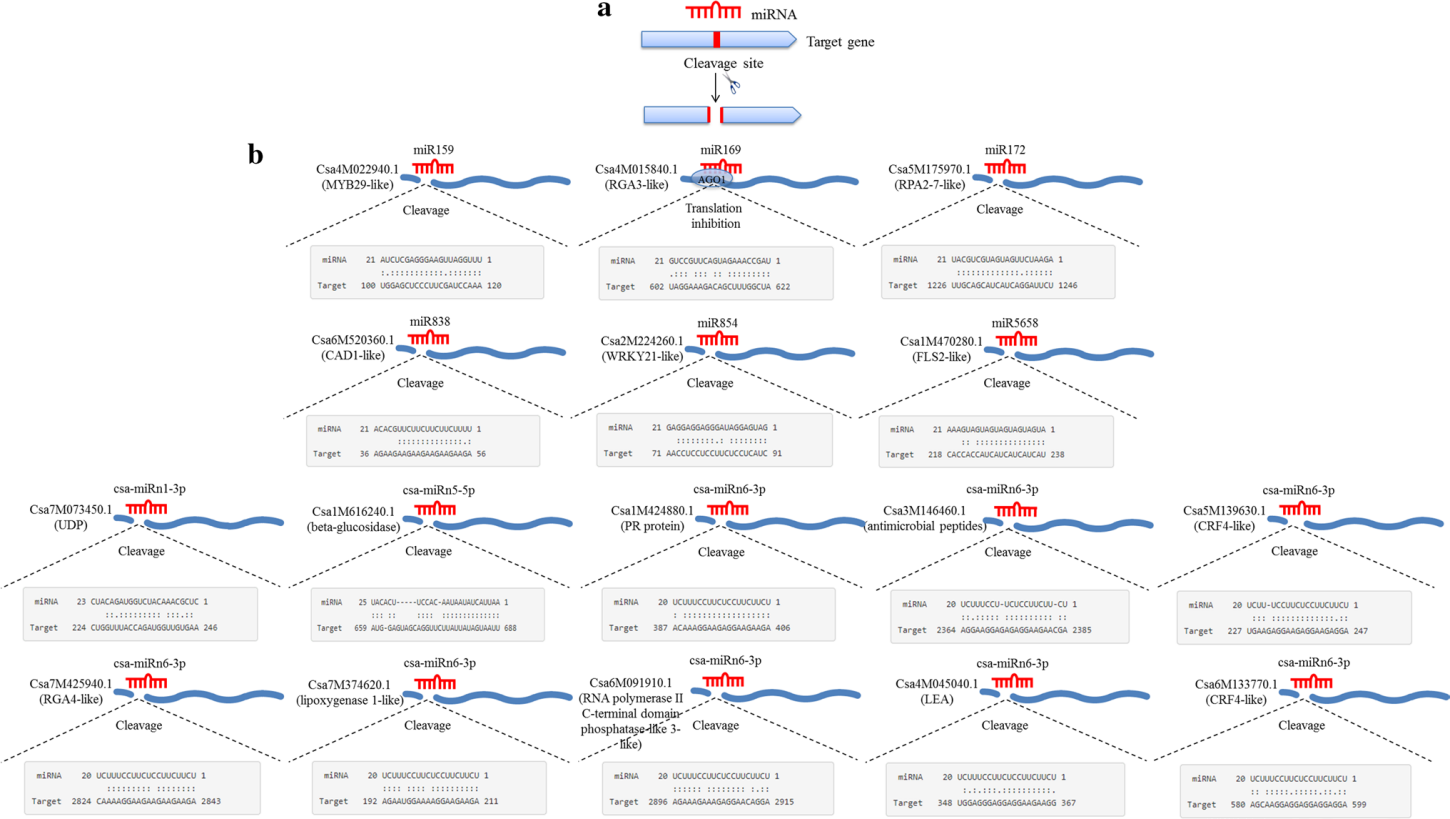
Predicted complementary sequences of miRNA and target genes in cucumber. **a** Diagram illustrating miRNA-mediated cleavage of target mRNAs. **b** Alignment of specific miRNA-target mRNA duplexes. Cleavage is typically more efficient in the absence of a central mismatch, whereas translational inhibition is facilitated by the presence of a mismatch [37, 38]

It is well established that miRNAs frequently function by repressing the expression of their target genes via matching of short complementary nucleotide sequences, where the miRNA-directed RNA-induced silencing complex (RISC) cleaves the target mRNA [45]. All of the 14 miRNAs examined in this study were found to contain characteristic complementary sequences that matched their target genes, and with the exception of miR169, which suppressed translation, all were found to be associated with mRNA cleavage, indicating that targeted degradation is the primary mode of miRNA regulation in cucumber (Fig. 2 and Table S3).

### Differential expression of cucumber miRNAs during CGMMV infection

**Fig. 3:**
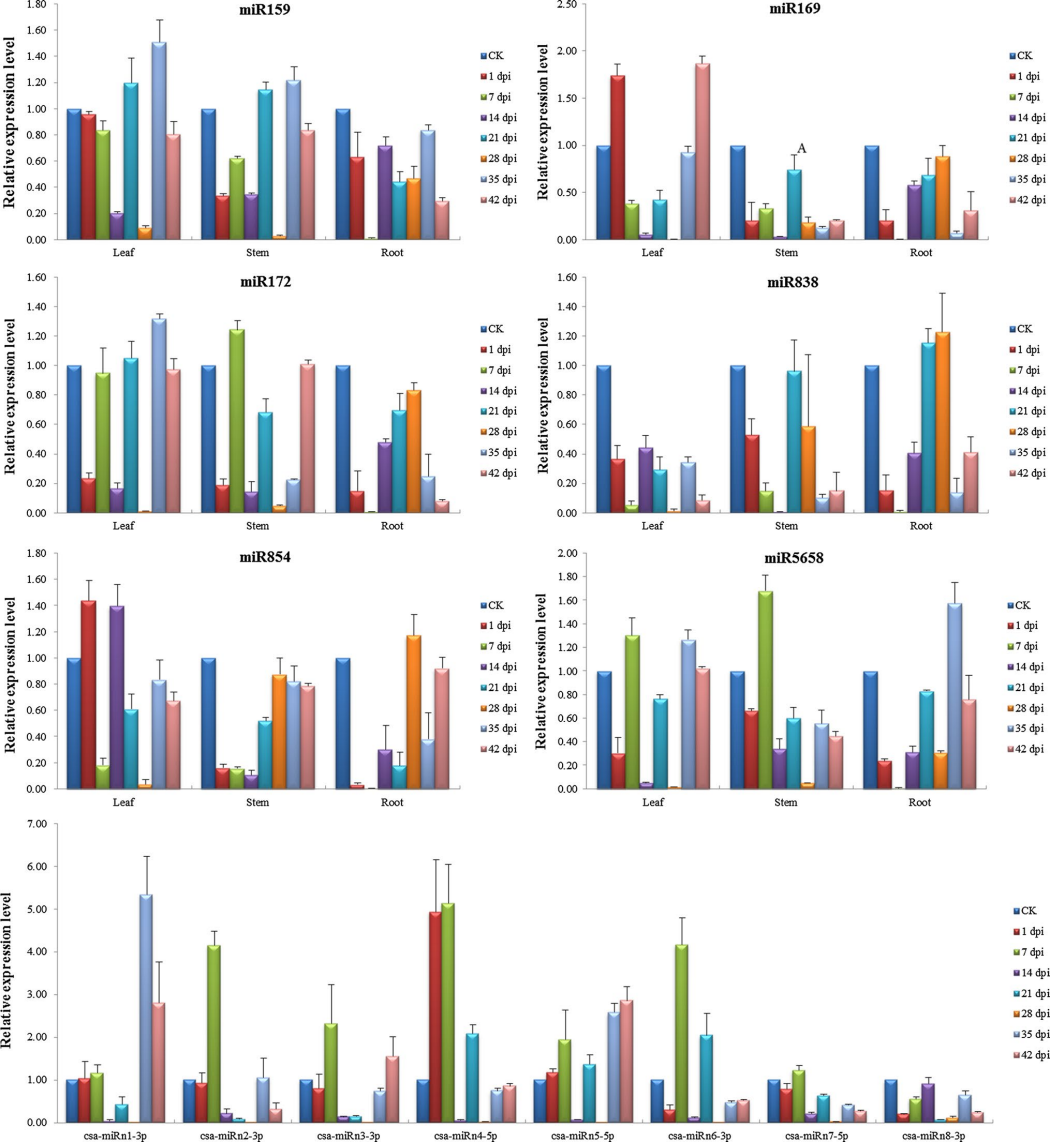
Relative expression levels of 14 miRNAs in different cucumber tissues at seven time points after inoculation with CGMMV. Expression levels of six known miRNAs in three different cucumber samples including leaves, stems and roots, as well as the expression of eight novel miRNAs in cucumber leaf samples are shown. The novel miRNAs were isolated from the leaf samples of CGMMV-infected cucumbers in a previous study [36]. Relative expression values were normalized using *EF-1α* and *Ubiquitin* as internal controls and standardized relative to the control (CK) values. Bars correspond to standard errors. dpi indicates the number of days post-inoculation

The expression levels of all of the 14 miRNAs were found to be significantly altered in CGMMV-infected cucumber plants (Fig. 3). However, the patterns of expression were extremely complex and varied according to the tissue type and time point post-inoculation. For example, miR169, miR854 and csa-miRn4-5p were significantly upregulated in leaf tissue at 1 dpi but downregulated at 28 dpi, while miR172, miR838, miR5658, csa-miRn6-3p and csa-miRn8-3p exhibited the opposite pattern of expression, being significantly downregulated at 1 dpi. In cucumber stem and root samples, six known miRNAs (miR159, miR169, miR172, miR838, miR854 and miR5658) were downregulated at 1 dpi. In cucumber leaf samples, significant upregulation was observed with csa-miRn1-3p at 35 dpi, csa-miRn2-3p at 7 dpi, csa-miRn3-3p at 7 dpi and csa-miRn5-5p between 35 and 42 dpi. The expression levels of csa-miRn7-5p and csa-miRn8-3p were always low compared with the other miRNAs.

### Differential expression of target genes during CGMMV infection

**Fig. 4:**
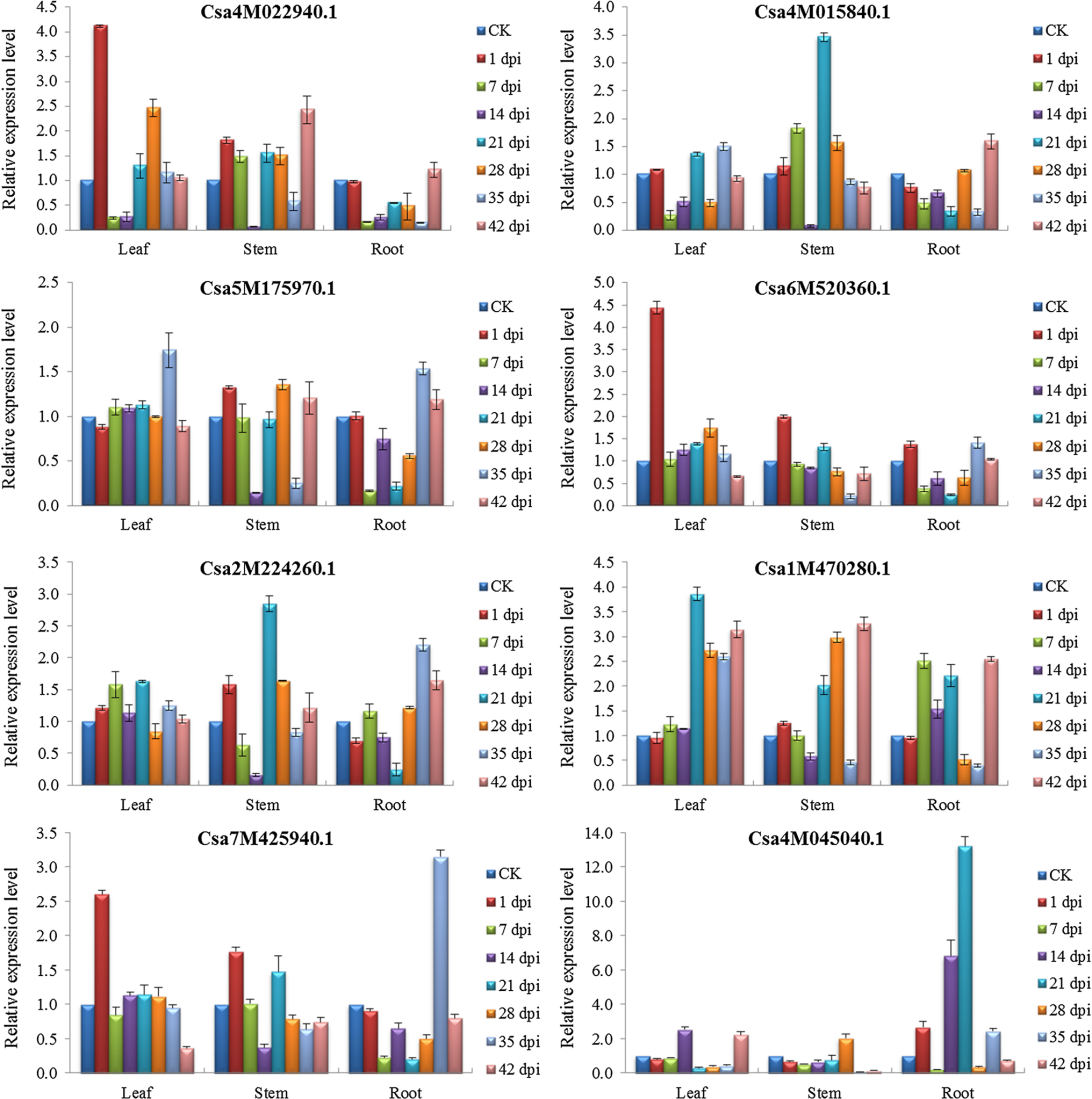
Relative expression levels of eight putative target genes associated with six known and one novel miRNAs in CGMMV-infected cucumber samples. Relative expression values were normalized using *EF-1α* and *Ubiquitin* as internal controls and standardized relative to the control (CK) values. Bars correspond to standard errors. dpi indicates the number of days post-inoculation

The expression levels of the eight target genes associated with plant defense were also altered in the CGMMV-infected samples and exhibited similarly complex patterns of expression (Fig. 4). For example, Csa4M022940.1 and Csa6M520360.1 were highly upregulated in leaf samples at 1 dpi, and Csa5M175970.1 was upregulated in leaf and root samples at 35 dpi. Csa1M470280.1 was dramatically upregulated in leaf samples during the later stages of infection (21-42 dpi), while Csa4M045040.1 was upregulated in root samples between 14 and 21 dpi. The expression of miR169 target Csa4M015840.1 (putative disease resistance protein RGA3-like) and miR854 target Csa2M224260.1 (probable WRKY transcription factor that plays an important role in plant defense responses) were highest at 21 dpi in stem samples. In addition, it was also noted that the expression level of csa-miRn6-3p target Csa7M425940.1 (putative disease resistance protein RGA4-like) at 1 dpi was considerably higher than at other time points in both the leaf and stem samples.

### Correlation between miRNA and target gene expression

The expression of miR172 exhibited a positive correlation with its target gene Csa5M175970.1 in cucumber leaf samples throughout the entire period of infection (1 to 42 dpi). However, in general, the correlation between the expression of miRNAs, including miR159, miR169, miR838, miR854, miR5658, csa-miRn6-3p, and their respective target genes varied depending on the time point, indicating that additional factors may be involved in regulating these target genes and that miRNA-target gene interactions are extremely complex (Table 2).

**Table 2:** Correlations between the expression of seven cucumber miRNAs and their target genes during CGMMV infection

miRNA/ target	Leaf							Stem							Root						
	1	7	14	21	28	35	42	1	7	14	21	28	35	42	1	7	14	21	28	35	42
miR159/Csa4M022940.1	**-**	**+**	**-**	**+**	**-**	**-**	**+**	**-**	**-**	**+**	**+**	**+**	**-**	**-**	**+**	**+**	**+**	**-**	**-**	**-**	**-**
miR169/Csa4M015840.1	**+**	**+**	**-**	**+**	**+**	**+**	**-**	**-**	**+**	**+**	**+**	**+**	**+**	**-**	**+**	**+**	**+**	**-**	**-**	**+**	**+**
miR172/Csa5M175970.1	**+**	**+**	**+**	**+**	**+**	**+**	**+**	**-**	**-**	**+**	**+**	**-**	**-**	**+**	**+**	**+**	**+**	**-**	**+**	**-**	**+**
miR838/Csa6M520360.1	**-**	**+**	**+**	**-**	**-**	**-**	**+**	**-**	**+**	**+**	**+**	**+**	**+**	**+**	**-**	**+**	**+**	**-**	**+**	**-**	**-**
miR854/Csa2M224260.1	**+**	**-**	**-**	**-**	**+**	**+**	**+**	**-**	**-**	**+**	**+**	**-**	**+**	**-**	**+**	**-**	**-**	**+**	**+**	**-**	**-**
miR5658/Csa1M470280.1	**+**	**+**	**+**	**+**	**+**	**-**	**-**	**-**	**-**	**+**	**+**	**-**	**-**	**-**	**+**	**-**	**-**	**+**	**+**	**-**	**-**
csa-miRn6-3p/Csa7M425940.1	**-**	**-**	**-**	**+**	**+**	**+**	**+**	No data													
csa-miRn6-3p/Csa4M045040.1	**+**	**-**	**-**	**-**	**+**	**+**	**-**														

Note: ‘1, 7, 14, 21, 28, 35, 42’ indicate the number of days post inoculation (dpi); ‘+’ indicates positive correlation, ‘-’ indicates negative correlation; Results of expression correlation between csa-miRn6-3p and its target genes during CGMMV infection were only available for the leaf sample as this novel miRNA was only found in the leaves of CGMMV-infected cucumbers

### Hypothetical model of miRNA-mediated regulation

**Fig. 5:**
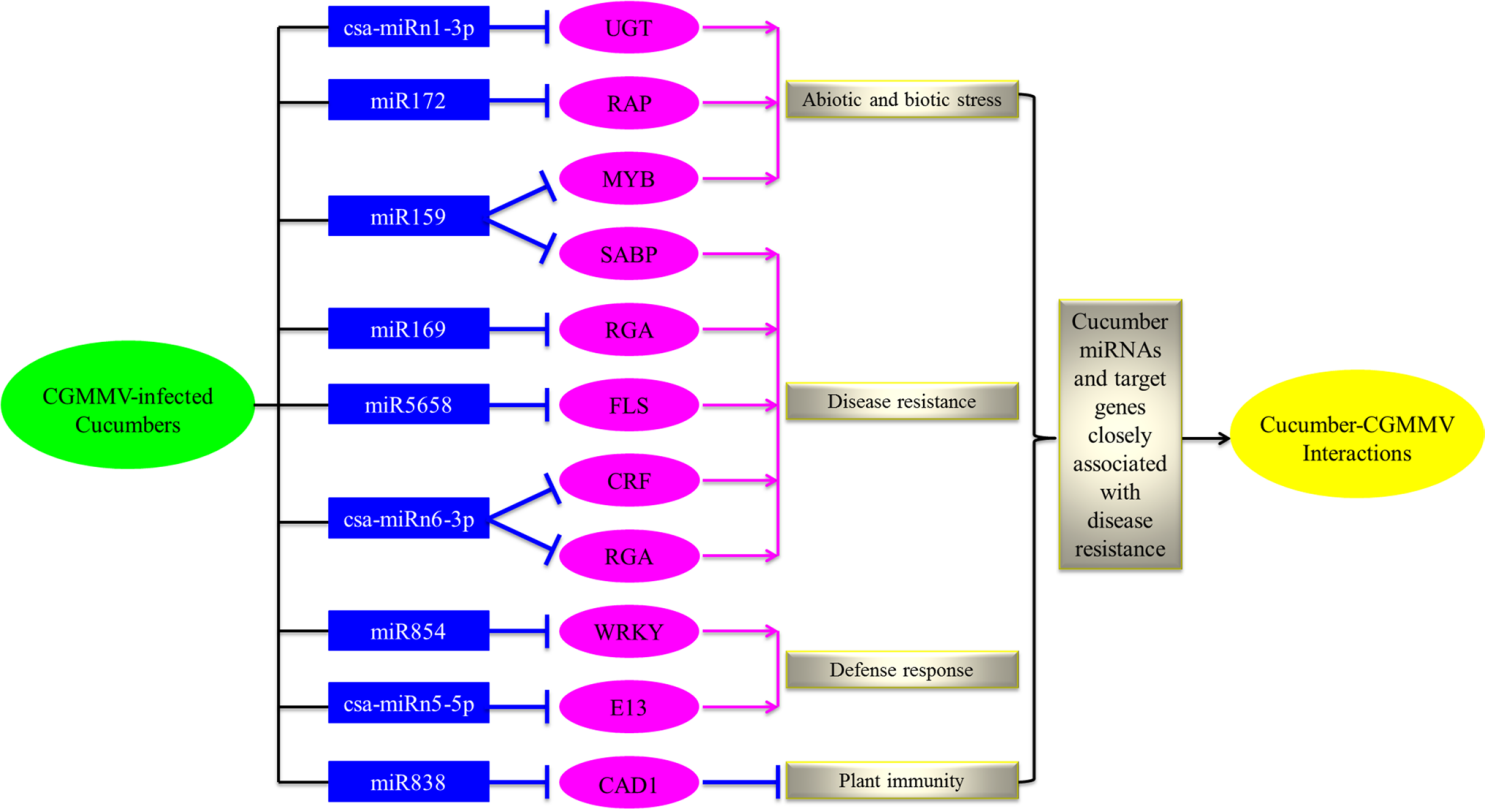
Hypothetical model of miRNA-mediated regulation of target genes in cucumber-CGMMV interactions. Pink arrows indicate positive regulation, and blue T shapes indicate negative regulation. UGT, uridine diphosphate glycosyltransferase; RAP, ethylene-responsive transcription factor RAP2-7; MYB, v-myb avian myeloblastosis viral oncogene homolog; SABP, salicylic acid (SA)-binding protein; RGA, resistance gene analogs; FLS, flavonol synthase; CRF, corticotropin-releasing factor; WRKY, WRKY transcription factor; E13, glucan endo-1,3-beta-glucosidase, basic isoform-like; CAD1, constitutively activated cell death 1

The regulatory network, functional allocation of putative target genes, and expression profile data were used to construct a hypothetical model that illustrates how nine miRNAs (miR159, miR169, miR172, miR838, miR854, miR5658, csa-miRn1-3p, csa-miRn5-5p and csa-miRn6-3p) interact with their target genes to influence biological processes associated with the cucumber-CGMMV pathosystem (Fig. 5). Most of the miRNAs influenced a single process. For example, miR172 and csa-miRn1-3 were associated with responses to abiotic and biotic stress, respectively. In this case, the target gene of miR172, the ethylene-responsive transcription factor RAP2-7, is known to regulate downstream gene expression in response to stress factors and components of stress signal transduction pathways. The target gene of csa-miRn1-3 was confirmed to be uridine diphosphate glycosyltransferase (UGT), which is strongly induced by H_2_O_2_,and this may interact with pathways associated with reactive oxygen species (ROS) and auxin signaling and play important roles in stress responses mediated by glycosylating hormones and secondary metabolites. In contrast, miR159 also plays an important role during abiotic and biotic stress and disease resistance via two target genes, an MYB transcription factor, which is known to be a positive regulator of abiotic stress tolerance and biotic stress resistance, and the salicylic acid (SA)-binding protein (SABP), which has been linked to disease resistance. Csa-miRn6-3p also regulates two target genes, corticotropin-releasing factor (CRF) and resistant gene analog (RGA), both of which are linked to disease resistance. Two other miRNAs also influenced disease resistance. The target gene of miR169 was identified as an RGA that can trigger a defense system that restricts the growth of pathogens. In addition, miR838 targeted an MACPF-domain-containing CAD1-like protein that negatively controls the SA-mediated pathway associated with programmed cell death during the hypersensitivity response [46, 47]. The target genes of miR854 and miR5658 are a probable WRKY transcription factor 21-like protein and LRR receptor-like serine/threonine-protein kinase FLS2-like protein, both of which are known to play an important role in defense responses and disease resistance in plant-pathogen interactions [48–53]. The target gene of csa-miRn5-5p is a glucan endo-1,3-beta-glucosidase (E13) that triggers a plant-defense-related product in response to fungal pathogens [54]. The hypothetical model demonstrates that a multitude of miRNAs and target genes can influence different metabolic pathways and cellular processes, the combined effect of which allows cucumber to cope with viral infection-related stress (Fig. 5).

## Discussion

MicroRNAs are small, non-coding RNAs that regulate gene expression in a sequence-specific manner [55]. To date, thousands of miRNAs have been identified in a diverse range of plants and other organisms. Many miRNAs exhibit complex patterns of expression and frequently regulate a variety of biological processes through miRNA-target mRNA interactions, including development, signal transduction, responses to environmental stress, and host-pathogen interactions [27]. Some miRNAs are known to play distinct roles in different genetic contexts. For example, in carrot plants, miR172 targets a G-box binding factor and the ABA response element-binding factor 1, while miR854 and miR5658 were found to target a symbiosis-related disease resistance protein and lateral suppressor-like protein, which are involved in responses to stress and growth and development, respectively [56]. However, in ginger, miR854 and another miRNA, miR838, were found to regulate rhizome development and the biosynthesis of essential oils [57]. Singh et al. [58] found that miR854 formed a regulatory network in association with miR5658 affecting a collection of shared target genes (Bra006283, Bra011043, Bra030266, Bra034179, Bra005011, and Bra001582) involved in transmembrane transport and zinc ion binding in *Brassica juncea*. Cucumber miR5658 was found to target Dof (DNA-binding with one finger) genes that encode plant-specific transcription factors that promote large-scale expression of defence-related genes that play significant roles in plant growth, development, and responses to biotic and abiotic stresses [59]. Mao et al. [60] found that 21 target mRNAs of 11 known miRNAs (miR156/157, miR159, miR164, miR167, miR169, miR172, miR319, miR393, miR398 and miR858) take part in a broad range of physiological processes, including transcription regulation, cell differentiation, organismal development, vegetative-to-reproductive phase transition, photosynthesis, defense against stresses, hormone stimulus, and light signaling pathways. In this study, functional analysis of the target genes in the network indicated that miR159, miR838, miR854, miR5658 and csa-miRn6-3p play a major role in the regulation of processes associated with the cucumber-CGMMV pathosystem.

Most of the miRNAs in this study were also found to affect multiple target genes (up to 196 in the case of csa-miRn6-3p). Furthermore, it was found that miR159, miR838, miR854 and miR5658 as well as csa-miRn6-3p were linked by a few shared target genes to form a large regulatory network that impacts 578 target genes. These results highlighted the interconnected nature of miRNA-target genes, where one gene can be controlled by more than one miRNA. This has also been shown by John et al. [61].

Assessing the expression level of miRNAs is an important step in understanding their biogenesis, function and regulatory mechanism. Several studies have shown that expression profiles and the abundance of miRNAs can be affected by virus infection, for example in rice infected with rice stripe virus (RSV) [62] and in watermelon and cucumber infected with CGMMV [35, 36]. This study showed that the expression levels of 14 miRNAs (miR159, miR169, miR172, miR838, miR854, miR5658, csa-miRn1-3p, csa-miRn2-3p, csa-miRn3-3p, csa-miRn4-5p, csa-miRn5-5p, csa-miRn6-3p, csa-miRn7-5p and csa-miRn8-3p) were dramatically affected by CGMMV infection and should therefore be considered CGMMV-responsive miRNAs in cucumber. It is interesting to note that many of these miRNAs have also been implicated in the pathology of virus infections in other plant species. For example, Naqvi et al. [63] observed the downregulation of miR169 and the upregulation of miR172 and miR159 in response to tomato leaf curl New Delhi virus (ToLCNDV) infection using microarray analysis and northern blotting, while Sun et al. [35] found that miR172 and miR838 were significantly affected by CGMMV infection. However, a genome-wide profiling study of drought-stressed rice showed that miR159 and miR172 were downregulated and that miR854 was upregulated [64]. Taken together, these results indicate that the CGMMV-responsive miRNAs identified in the current study of cucumber can have different roles in different genetic and environmental contexts, and they reaffirm the observation of Sun et al. [35] that miRNAs are expressed in a species-specific manner during virus infection, and therefore, their expression patterns should be studied individually.

The RT-qPCR analysis of target genes conducted in this study failed to show a consistent trend of expression during the course of CGMMV infection spanning from 1 to 42 dpi. The expression of target genes could have been influenced by miRNA abundance in a spatiotemporal and tissue-specific manner. A previous study of cucumber miRNAs in response to CGMMV infection suggested that the expression of target genes may be restricted in specific tissues or at particular developmental stages, and it was concluded that this was an indication that miRNAs are not the only factors influencing target genes [65]. In addition, differences in viral silencing suppressor activity over the course of an infection are also likely to have influenced target genes related to resistance.

Plant miRNAs most commonly exert negative regulation of their target genes via post-transcriptional cleavage of target mRNAs, a process that is catalyzed by the RNA-induced silencing complex (RISC) and is mediated by complementary sequences in the miRNA and target gene [22, 66–69]. For instance, downregulation of miR398 in *Arabidopsis* leaves results in upregulation of its Cu/Zn superoxide dismutase target gene, which is involved in the defense response to *Pseudomonas syringae* [70]. However, there have also been reports of miRNAs and their target genes being positively correlated. For example, Kawashima et al. [71] discovered that the expression of both miR395 and its target gene, sulphate transporter (SULTR2;1) were highly elevated in the roots of *Arabidopsis* plants in response to sulphur starvation. It is therefore interesting to note that we found a positive correlation between the expression of miR172 and its target Csa5M175970.1 (ethylene-responsive transcription factor RAP2-7) in the leaves of cucumber throughout the entire period of CGMMV infection (1 to 42 dpi). However, in most cases it was difficult to identify clear correlations between the other cucumber miRNAs and their respective target genes, which again suggests that some additional factors may influence cucumber miRNA-target interactions and highlights the complexity of the regulatory network. Additional studies are required to further characterize the interplay of cucumber miRNA expression and regulation and its effect on downstream biological processes.

## Electronic supplementary material

Below is the link to the electronic supplementary material.

Supplementary material 1 (XLS 29 kb)

Supplementary material 2 (XLS 31 kb)

Supplementary material 3 (XLS 155 kb)

Supplementary material 4 (DOC 393 kb)

Supplementary material 5 (DOCX 931 kb)

Supplementary material 6 (DOCX 294 kb)

## Abbreviations

CGMMV: Cucumber green mottle mosaic virus
DAS-ELISA: Double antibody sandwich enzyme-linked immunosorbent assay
dpi: Days post-inoculation
GO: Gene Ontology
KEGG: Kyoto Encyclopedia of Genes and Genomes
NCBI: National Center for Biotechnology Information
psRNATarget: A Plant Small RNA Target Analysis Server
RT-PCR: Reverse transcription polymerase chain reaction
RT-qPCR: Reverse transcription quantitative real-time PCR
RISC: RNA-induced silencing complex
VSRs: Viral suppressors of RNA silencing
