# Supplementary material for: Expression profiling and regulatory network of cucumber microRNAs and their putative target genes in response to cucumber green mottle mosaic virus infection
Source: Arch Virol. 2019 Feb 24;164(4):1121–34. doi: 10.1007/s00705-019-04152-w (PMC6420491; doi:10.1007/s00705-019-04152-w)
Supplement: Supplementary file 4 — Supplementary material 4 (DOC 393 kb) [file 705_2019_4152_MOESM4_ESM.doc]

Supplementary material 4 Table S4 Function annotation of target genes of cucumber novel miRNAs.

| **No.** | **miRNA Acc.** | **Target Acc.** | **Target gene** | **GenBank Accession** | **Function annotation** | **Expectation** | **UPE** | **Inhibition** |
| --- | --- | --- | --- | --- | --- | --- | --- | --- |
| 1 | csa-miRn1-3p | Csa6M505970.1 | uncharacterized | XM004158818.1  XM004134810.1 | - | 2 | 20.034 | Cleavage |
| 2 | csa-miRn1-3p | Csa7M073450.1 | UDP-glycosyltransferase 73B3-like | XR180760.1 | Pathogen-Responsive Expression of Glycosyltransferase  Genes UGT73B3 and UGT73B5 Is Necessary for  Resistance to *Pseudomonas syringae* pv tomato  in *Arabidopsis* | 3 | 16.702 | Cleavage |
| 3 | csa-miRn1-3p | Csa3M854260.1 | putative pentatricopeptide repeat-containing protein At1g19290-like | XM004147769.1  XM004154856.1  XM004172425.1 | - | 3 | 19.477 | Cleavage |
| 4 | csa-miRn2-3p | Csa6M488360.1 | BTB/POZ domain-containing protein At5g66560-like, transcript variant 2 | XM004160447.1  XM004141897.1 | Expression of the BTB/POZ domain-containing protein At1g63850-like gene CsFDI1 is enhanced by sugar starvation in cucumber fruit | 2.5 | 18.889 | Cleavage |
|  |  |  | BTB/POZ domain-containing protein At5g66560-like, transcript variant 1 | XM004160446.1  XM004141896.1 |  |  |  |  |
| 5 | csa-miRn3-3p | Csa5M166410.2 | 60S ribosomal protein L36-2-like | XM004152333.1 | Gene:RPL36b; Organism: *Arabidopsis thaliana*; Molecular function: structural constituent of ribosome | 3 | 16.233 | Translation |
| 6 | csa-miRn3-3p | Csa5M166410.1 | 60S ribosomal protein L36-2-like | XM004152333.1 |  | 3 | 16.233 | Translation |
| 7 | csa-miRn4-5p | Csa3M901140.1 | - | - | - | 2 | 20.2 | Cleavage |
| 8 | csa-miRn5-5p | Csa3M912290.1 | uncharacterized | XM004148322.1  XM004167073.1  XM004165058.1  XM004172098.1 | - | 2 | 18.135 | Cleavage |
| 9 | csa-miRn5-5p | Csa1M616240.1 | glucan endo-1,3-beta-glucosidase, basic isoform-like | XM004139071.1  XM004138998.1 | Gene:GLUB1; Organism: *Solanum tuberosum* (Potato); Function: Is thought to be an important plant defense-related product against fungal pathogens. | 2.5 | 11.703 | Cleavage |
| 10 | csa-miRn5-5p | Csa3M019350.1 | putative dual specificity protein phosphatase DSP8-like | XM004168878.1  XM004147173.1 | Gene:DSP8; Organism: *Arabidopsis thaliana*; Function: May possess phosphatase activity. | 3 | 15.573 | Cleavage |
| 11 | csa-miRn6-3p | Csa3M019980.1 | zinc finger protein ZAT9-like | XM004147206.1 | - | 0.5 | 7.815 | Cleavage |
| 12 | csa-miRn6-3p | Csa2M380020.2 | uncharacterized | XM004138818.1  XM004172633.1 | - | 1 | 6.056 | Cleavage |
|  | csa-miRn6-3p | Csa2M380020.2 | peptidyl-prolyl cis-trans isomerase cyp5-like | XM004164484.1 | Gene:cyp5; Organism: *Rhizopus delemar (strain RA 99-880 / ATCC MYA-4621 / FGSC 9543 / NRRL 43880) (Mucormycosis agent) (Rhizopus arrhizus var. delemar)*; Function: PPIases accelerate the folding of proteins. It catalyzes the cis-trans isomerization of proline imidic peptide bonds in oligopeptides (By similarity). |  |  |  |
| 13 | csa-miRn6-3p | Csa2M380020.4 | uncharacterized | XM004138818.1  XM004172633.1 | - | 1 | 6.056 | Cleavage |
|  | csa-miRn6-3p | Csa2M380020.4 | peptidyl-prolyl cis-trans isomerase cyp5-like | XM004164484.1 | Gene:cyp5; Organism: *Rhizopus delemar (strain RA 99-880 / ATCC MYA-4621 / FGSC 9543 / NRRL 43880) (Mucormycosis agent) (Rhizopus arrhizus var. delemar)*; Function: PPIases accelerate the folding of proteins. It catalyzes the cis-trans isomerization of proline imidic peptide bonds in oligopeptides (By similarity). |  |  |  |
| 14 | csa-miRn6-3p | Csa2M380020.1 | peptidyl-prolyl cis-trans isomerase cyp5-like | XM004164484.1 |  | 1 | 6.056 | Cleavage |
| 15 | csa-miRn6-3p | Csa2M380020.3 | peptidyl-prolyl cis-trans isomerase cyp5-like | XM004164484.1 |  | 1 | 6.056 | Cleavage |
|  | csa-miRn6-3p | Csa2M380020.3 | uncharacterized | XM004138818.1  XM004172633.1 | - |  |  |  |
| 16 | csa-miRn6-3p | Csa3M119700.1 | probable WRKY transcription factor 41-like, misc RNA | XR180939.1  XR18074.1 | Gene:WRKY41; Organism: *Arabidopsis thaliana*; Function: Transcription factor. Interacts specifically with the W box (5'-(T)TGAC[CT]-3'), a frequently occurring elicitor-responsive cis-acting element (By similarity). | 1 | 21.965 | Cleavage |
|  | csa-miRn6-3p | Csa3M119700.1 | clon CU16D01 putative WRKY transcription factor 30 mRNA, partial cds | FJ036895.1 | Gene:N/A; Organism: *Cucumis sativus (Cucumber);* Function: sequence-specific DNA binding transcription factor activity. |  |  |  |
| 17 | csa-miRn6-3p | Csa3M690290.1 | DNA topoisomerase 2-like | XM004165336.1  XM004149668.1 | Protein:DNA topoisomerase 2-alpha; Gene:TOP2A; Organism: *Human;* Function: Control of topological states of DNA by transient breakage and subsequent rejoining of DNA strands. Topoisomerase II makes double-strand breaks. Essential during mitosis and meiosis for proper segregation of daughter chromosomes. May play a role in regulating the period length of ARNTL/BMAL1 transcriptional oscillation (By similarity). | 1.5 | 15.255 | Cleavage |
| 18 | csa-miRn6-3p | Csa4M000700.1 | telomere repeat-binding factor 1-like | XM004170684.1  XM004150209.1 | Exercise alters mRNA expression of telomere-repeat binding factor 1 in skeletal muscle via p38 MAPK | 1 | 6.028 | Cleavage |
| 19 | csa-miRn6-3p | Csa6M104100.1 | pre-mRNA-processing factor 6-like | XM004164407.1  XM004140467.1 | *STA1*, an *Arabidopsis* pre-mRNA processing factor 6 homolog, is a new player involved in miRNA biogenesis | 1.5 | 20.659 | Cleavage |
| 20 | csa-miRn6-3p | Csa7M357030.1 | transcription initiation factor TFIID subunit 1-A-like | XM004149737.1  XM004149737.1 | Protein:Transcription initiation factor TFIID subunit 1; Gene:TAF1; Organism:*Homo sapiens (Human)*; Function:Largest component and core scaffold of the TFIID basal transcription factor complex. Contains novel N- and C-terminal Ser/Thr kinase domains which can autophosphorylate or transphosphorylate other transcription factors. Phosphorylates TP53 on 'Thr-55' which leads to MDM2-mediated degradation of TP53. Phosphorylates GTF2A1 and GTF2F1 on Ser residues. Possesses DNA-binding activity. Essential for progression of the G1 phase of the cell cycle. Exhibits histone acetyltransferase activity towards histones H3 and H4. | 2 | 11.35 | Cleavage |
| 21 | csa-miRn6-3p | Csa1M424880.1 | pathogenesis-related homeodomain protein-like | XM004146323.1  XM004161754.1 | Gene:PRH; Organism: *Arabidopsis thaliana*; Function: Specifically binds to the fungal elicitor-responsive DNA element, 5'-CTAATTGTTTA-3', of the gene PR2 promoter. GO-Biological process: defense response, response to auxin, transcription, DNA-templated | 1 | 7.351 | Cleavage |
| 22 | csa-miRn6-3p | Csa1M605660.1 | putative peptide/nitrate transporter At5g19640-like | XM004161531.1  XM004148985.1 | - | 1.5 | 11.186 | Cleavage |
| 23 | csa-miRn6-3p | Csa3M232950.1 | probable serine/threonine-protein kinase At1g54610-like | XM004146200.1 | *Arabidopsis thaliana*: ATP binding; protein serine/threonine kinase activity | 2 | 19.529 | Cleavage |
| 24 | csa-miRn6-3p | Csa6M502040.1 | - | - | - | 2 | 2.942 | Cleavage |
| 25 | csa-miRn6-3p | Csa5M608280.2 | protein CHUP1, chloroplastic-like | XM004135071.1  XM004159258.1 | Gene:CHUP1; Organism: *Arabidopsis thaliana*; Function: Required for the positioning and movement of chloroplasts. Interacts with profilin and actin independent of its polymerization status. Regulates chloroplast localization by anchoring chloroplasts to the plasma membrane and forming a bridge to the actin cytoskeleton. | 2 | 13.917 | Cleavage |
| 26 | csa-miRn6-3p | Csa5M608280.1 | protein CHUP1, chloroplastic-like | XM004135071.1  XM004159258.1 |  | 2 | 13.917 | Cleavage |
| 27 | csa-miRn6-3p | Csa6M361300.1 | - | - | - | 2 | 5.955 | Cleavage |
| 28 | csa-miRn6-3p | Csa1M144290.1 | vacuolar protein sorting-associated protein 41 homolog | XM004139411.1 | Gene:VPS41; Organism: *Solanum lycopersicum (Tomato) (Lycopersicon esculentum)*; Function: Required for vacuolar assembly and vacuolar traffic. | 2 | 9.837 | Cleavage |
| 29 | csa-miRn6-3p | Csa6M079210.1 | uncharacterized | XM004145025.1 | - | 1 | 8.231 | Translation |
| 30 | csa-miRn6-3p | Csa2M020850.1 | - | - | - | 2 | 1.594 | Cleavage |
| 31 | csa-miRn6-3p | Csa6M425750.1 | uncharacterized | XM004140215.1  XM004173717.1 | - | 2 | 22.992 | Cleavage |
| 32 | csa-miRn6-3p | Csa6M425750.2 | uncharacterized | XM004140215.1  XM004173717.1 | - | 2 | 22.992 | Cleavage |
| 33 | csa-miRn6-3p | Csa6M516920.1 | sugar carrier protein C-like | XM004134683.1  XM004168740.1 | Protein: sugar carrier protein A; Organism:*Populus trichocarpa (Western balsam poplar) (Populus balsamifera subsp. trichocarpa)*; Molecular function: substrate-specific transmembrane transporter activity. | 2 | 10.54 | Cleavage |
| 34 | csa-miRn6-3p | Csa1M007860.1 | Serine/threonine-protein kinase AtPK2/AtPK19-like | XM004138089.1 | Gene:ATPK2; Organism: *Arabidopsis thaliana*; Function: Downstream effector of TOR signaling pathway. May be involved in adaptation of plant to cold or high-salt conditions. | 2 | 19.815 | Cleavage |
| 35 | csa-miRn6-3p | Csa2M013230.1 | translation initiation factor eIF-2B subunit epsilon-like | XM004139161.1 | Gene:EIF2B5; Organism: *Oryctolagus cuniculus (Rabbit)*; Function: Catalyzes the exchange of eukaryotic initiation factor 2-bound GDP for GTP. | 2.5 | 6.441 | Cleavage |
| 36 | csa-miRn6-3p | Csa1M423320.1 | U3 small nucleolar RNA-associated protein 25-like | XM004161753.1 | DEAD-box RNA helicase-like protein required for pre-18S rRNA processing, specifically at sites A0, A1, and A2. | 2.5 | 9.588 | Cleavage |
| 37 | csa-miRn6-3p | Csa3M912940.1 | bax inhibitor 1-like, transcript variant 2 | XM004148314.1 | - | 2 | 16.809 | Cleavage |
|  |  |  | bax inhibitor 1-like, transcript variant 1 | XM004148313.1 |  |  |  |  |
|  | csa-miRn6-3p | Csa3M912940.1 | BAX inhibitor-1 (BI-1), partial cds | KC514354.1 |  |  |  |  |
|  | csa-miRn6-3p | Csa3M912940.1 | bax inhibitor 1-like | XM004148312.1 |  |  |  |  |
| 38 | csa-miRn6-3p | Csa3M118730.1 | uncharacterized | XM004167076.1  XM004134245.1 | - | 2.5 | 6.745 | Cleavage |
| 39 | csa-miRn6-3p | Csa3M182050.1 | O-glucosyltransferase rumi homolog | XM004140796.1  XM004168487.1 | transferase activity, transferring glycosyl groups | 1.5 | 20.326 | Cleavage |
| 40 | csa-miRn6-3p | Csa1M097640.1 | - | - | - | 1.5 | 4.004 | Cleavage |
| 41 | csa-miRn6-3p | Csa5M139460.1 | uncharacterized | XM004145937.1  XM004160357.1 | - | 2.5 | 2.256 | Cleavage |
| 42 | csa-miRn6-3p | Csa1M707120.1 | uncharacterized | XM004173897.1  XM004145432.1 | - | 2 | 4.72 | Cleavage |
| 43 | csa-miRn6-3p | Csa3M017060.1 | uncharacterized | XM004164570.1 | - | 2.5 | 0.388 | Cleavage |
| 44 | csa-miRn6-3p | Csa4M578870.1 | microfibrillar-associated protein 1-like | XM004167608.1  XM004144607.1 | Recombinant Human Microfibrillar-associated Protein 4, FLAG-tagged: This gene encodes a protein with similarity to a bovine microfibril-associated protein. The protein has binding specificities for both collagen and carbohydrate. It is thought to be an extracellular matrix protein which is involved in cell adhesion or intercellular interactions. The gene is located within the Smith-Magenis syndrome region. | 2.5 | 15.798 | Cleavage |
| 45 | csa-miRn6-3p | Csa3M829040.1 | putative cyclic nucleotide-gated ion channel 8-like | XM004147405.1  XM004171053.1 | Gene:CNGC8; Organism: *Arabidopsis thaliana*; Function: Putative cyclic nucleotide-gated ion channel. | 2.5 | 24.028 | Cleavage |
| 46 | csa-miRn6-3p | Csa6M525380.1 | FACT complex subunit SPT16-like | XM004134515.1  XM004158605.1 | Gene:SPT16; Organism: *Saccharomyces cerevisiae (strain ATCC 204508 / S288c) (Baker's yeast)*; Function: Component of the FACT complex, a general chromatin factor that acts to reorganize nucleosomes. The FACT complex is involved in multiple processes that require DNA as a template such as mRNA elongation, DNA replication and DNA repair. During transcription elongation the FACT complex acts as a histone chaperone that both destabilizes and restores nucleosomal structure. It facilitates the passage of RNA polymerase II and transcription by promoting the dissociation of one histone H2A-H2B dimer from the nucleosome, then subsequently promotes the reestablishment of the nucleosome following the passage of RNA polymerase II. Transcription elongation is promoted by the repression of transcription initiation from cryptic sites. Also acts in establishing transcription initiation complexes and promotes SPT15/TBP-binding to a TATA box. Together with replication factor-A protein (RPA), FACT may play a role in nucleosome deposition during DNA replication. | 2 | 8.231 | Cleavage |
| 47 | csa-miRn6-3p | Csa3M777630.1 | - | - | - | 2 | 12.906 | Cleavage |
| 48 | csa-miRn6-3p | Csa3M848220.1 | transcription factor tau subunit sfc4-like | XM004146801.1 | - | 2.5 | 6.056 | Cleavage |
|  | csa-miRn6-3p | Csa3M848220.1 | general transcription factor 3C polypeptide 3-like | XM004154749.1 | general transcription factor 3C polypeptide 6， Human, Involved in RNA polymerase III-mediated transcription. Integral, tightly associated component of the DNA-binding TFIIIC2 subcomplex that directly binds tRNA and virus-associated RNA promoters. |  |  |  |
| 49 | csa-miRn6-3p | Csa3M854250.1 | probable calcium-binding protein CML41-like | XM004147770.1 | Gene:CML41; Organism: *Arabidopsis thaliana*; Function: Potential calcium sensor. | 2 | 8.819 | Cleavage |
| 50 | csa-miRn6-3p | Csa5M430000.1 | exocyst complex component 7-like | XM004140302.1 | Protein:Exocyst complex component 8; Gene:Exoc8; Organism:*Rattus norvegicus* (Rat); Function: Component of the exocyst complex involved in the docking of exocytic vesicles with fusion sites on the plasma membrane. | 2 | 10.036 | Cleavage |
| 51 | csa-miRn6-3p | Csa6M486730.1 | RNA-binding protein 39-like | XM004141929.1  XM004160434.1 | - | 2.5 | 0.839 | Cleavage |
| 52 | csa-miRn6-3p | Csa3M852500.1 | uncharacterized | XM004147783.1 | - | 3 | 14.362 | Cleavage |
| 53 | csa-miRn6-3p | Csa3M009460.1 | uncharacterized | NM001280609.1 | - | 2 | 5.577 | Translation |
| 54 | csa-miRn6-3p | Csa2M432220.1 | cytochrome P450 89A2-like | XM004156736.1 | - | 2 | 14.548 | Cleavage |
| 55 | csa-miRn6-3p | Csa5M638410.1 | uncharacterized | XM004156116.1  XM004141416.1 | - | 2 | 10.454 | Translation |
| 56 | csa-miRn6-3p | Csa7M451960.1 | uncharacterized | XM004155927.1  XM004141754.1 | - | 3 | 7.723 | Cleavage |
| 57 | csa-miRn6-3p | Csa6M106760.1 | uncharacterized | XM004165916.1  XM004140459.1 | - | 2.5 | 11.205 | Cleavage |
| 58 | csa-miRn6-3p | Csa3M881590.1 | DDB1- and CUL4-associated factor homolog 1-like | XM004162451.1  XM004136411.1 | Gene:DCAF1; Organism: *Arabidopsis thaliana*; Function: Component of the CUL4-RBX1-DDB1-DCAF1 E3 ubiquitin-protein ligase complex, DCAF1 may function as the substrate recognition module within this complex. Appears to be required for plant embryogenesis and to affect several other developmental processes including leaf, shoot, and flower development. | 3 | 21.93 | Cleavage |
| 59 | csa-miRn6-3p | Csa3M116810.2 | protein IQ-DOMAIN 14-like | XM004155152.1  XM004134233.1 | Gene:IQD14; Organism: *Arabidopsis thaliana*; Function: May be involved in cooperative interactions with calmodulins or calmodulin-like proteins. May associate with nucleic acids and regulate gene expression at the transcriptional or post-transcriptional level (By similarity). | 2.5 | 15.566 | Cleavage |
| 60 | csa-miRn6-3p | Csa3M116810.1 | protein IQ-DOMAIN 14-like | XM004155152.1  XM004134233.1 |  | 2.5 | 15.566 | Cleavage |
| 61 | csa-miRn6-3p | Csa3M236020.1 | uncharacterized | XM004146227.1  XM004166299.1 | - | 3 | 2.162 | Cleavage |
| 62 | csa-miRn6-3p | Csa3M225830.1 | uncharacterized | XM004146206.1  XM004159822.1  XM004172958.1 | - | 2 | 6.456 | Translation |
| 63 | csa-miRn6-3p | Csa1M008440.1 | uncharacterized | XM004138095.1 | - | 3 | 9.697 | Cleavage |
| 64 | csa-miRn6-3p | Csa5M643360.1 | uncharacterized | XM004141440.1  XM004156182.1 | - | 2 | 13.774 | Cleavage |
| 65 | csa-miRn6-3p | Csa7M065120.1 | protein EXECUTER 1, chloroplastic-like | XM004137014.1  XM004165319.1 | Gene:EX1; Organism: *Arabidopsis thaliana*; Function: Together with EX2, enables higher plants to perceive singlet oxygen as a stress signal in plastid that activates a genetically determined nuclear stress response program which triggers a programmed cell death (PCD). This transfer of singlet oxygen-induced stress-related signals from the plastid to the nucleus that triggers genetically controlled PCD pathway is unique to photosynthetic eukaryotes and operates under mild stress conditions, impeding photosystem II (PSII) without causing photooxidative damage of the plant. (GO - Biological process: response to singlet oxygen; singlet oxygen-mediated programmed cell death) | 3 | 2.143 | Cleavage |
| 66 | csa-miRn6-3p | Csa7M065120.2 | protein EXECUTER 1, chloroplastic-like | XM004137014.1  XM004165319.1 |  | 3 | 2.143 | Cleavage |
| 67 | csa-miRn6-3p | Csa7M065120.3 | protein EXECUTER 1, chloroplastic-like | XM004137014.1  XM004165319.1 |  | 3 | 2.143 | Cleavage |
| 68 | csa-miRn6-3p | Csa4M290190.1 | uncharacterized | XM004142181.1 | - | 3 | 0.855 | Cleavage |
|  | csa-miRn6-3p | Csa4M290190.1 | UDP-glycosyltransferase 92A1-like | XM004162052.1 | - |  |  |  |
| 69 | csa-miRn6-3p | Csa6M043490.1 | uncharacterized | XM004140843.1  XM004173361.1 | - | 3 | 4.989 | Cleavage |
| 70 | csa-miRn6-3p | Csa1M366960.1 | uncharacterized | XM004169933.1  XM004148670.1 | - | 2.5 | 23.036 | Cleavage |
| 71 | csa-miRn6-3p | Csa3M736920.1 | DEAD-box ATP-dependent RNA helicase 57-like | XM004137763.1  XM004165100.1  XM004166320.1 | - | 3 | 17.602 | Cleavage |
| 72 | csa-miRn6-3p | Csa3M736920.2 | DEAD-box ATP-dependent RNA helicase 57-like | XM004137763.1  XM004165100.1  XM004166320.1 |  | 3 | 17.602 | Cleavage |
| 73 | csa-miRn6-3p | Csa6M157050.1 | uncharacterized | XM004149238.1  XM004162429.1 | - | 3 | 10.465 | Cleavage |
| 74 | csa-miRn6-3p | Csa3M129620.1 | uncharacterized | XM004174059.1  XM004133930.1 | - | 2.5 | 9.958 | Cleavage |
| 75 | csa-miRn6-3p | Csa3M207890.1 | ferredoxin--nitrite reductase, chloroplastic-like | XM004140647.1  XM004163867.1 | Gene:Os01g0357100; Organism:*Oryza sativa subsp. japonica (Rice)*; Function: Catalyzes the six-electron reduction of nitrite to ammonium. | 3 | 10.219 | Cleavage |
| 76 | csa-miRn6-3p | Csa4M188930.1 | exosome component 10-like | XM004149064.1  XM004168221.1 | Gene:Exosc10; Organism: *Mus musculus (Mouse)*; Function: Putative catalytic component of the RNA exosome complex which has 3'->5' exoribonuclease activity and participates in a multitude of cellular RNA processing and degradation events. In the nucleus, the RNA exosome complex is involved in proper maturation of stable RNA species such as rRNA, snRNA and snoRNA, in the elimination of RNA processing by-products and non-coding 'pervasive' transcripts, such as antisense RNA species and promoter-upstream transcripts (PROMPTs), and of mRNAs with processing defects, thereby limiting or excluding their export to the cytoplasm. The RNA exosome may be involved in Ig class switch recombination (CSR) and/or Ig variable region somatic hypermutation (SHM) by targeting AICDA deamination activity to transcribed dsDNA substrates. In the cytoplasm, the RNA exosome complex is involved in general mRNA turnover and specifically degrades inherently unstable mRNAs containing AU-rich elements (AREs) within their 3' untranslated regions, and in RNA surveillance pathways, preventing translation of aberrant mRNAs. It seems to be involved in degradation of histone mRNA. EXOSC10 has 3'-5' exonuclease activity (By similarity). EXOSC10 is required for nucleolar localization of C1D and probably mediates the association of SKIV2L2, C1D and MPP6 wth the RNA exosome involved in the maturation of 5.8S rRNA (By similarity) | 3 | 19.479 | Cleavage |
| 77 | csa-miRn6-3p | Csa3M859680.1 | uncharacterized | XM004147807.1  XM004154799.1 | - | 3 | 6.986 | Cleavage |
| 78 | csa-miRn6-3p | Csa6M185330.1 | protein DAMAGED DNA-BINDING 2-like | XM004143107.1  XM004160122.1 | DDB1; Human; Required for DNA repair. Binds to DDB2 to form the UV-damaged DNA-binding protein complex (the UV-DDB complex). The UV-DDB complex may recognize UV-induced DNA damage and recruit proteins of the nucleotide excision repair pathway (the NER pathway) to initiate DNA repair. The UV-DDB complex preferentially binds to cyclobutane pyrimidine dimers (CPD), 6-4 photoproducts (6-4 PP), apurinic sites and short mismatches. Also appears to function as a component of numerous distinct DCX (DDB1-CUL4-X-box) E3 ubiquitin-protein ligase complexes which mediate the ubiquitination and subsequent proteasomal degradation of target proteins. The functional specificity of the DCX E3 ubiquitin-protein ligase complex is determined by the variable substrate recognition component recruited by DDB1. DCX(DDB2) (also known as DDB1-CUL4-ROC1, CUL4-DDB-ROC1 and CUL4-DDB-RBX1) may ubiquitinate histone H2A, histone H3 and histone H4 at sites of UV-induced DNA damage. The ubiquitination of histones may facilitate their removal from the nucleosome and promote subsequent DNA repair. DCX(DDB2) also ubiquitinates XPC, which may enhance DNA-binding by XPC and promote NER. DCX(DTL) plays a role in PCNA-dependent polyubiquitination of CDT1 and MDM2-dependent ubiquitination of TP53 in response to radiation-induced DNA damage and during DNA replication. DCX(ERCC8) (the CSA complex) plays a role in transcription-coupled repair (TCR). May also play a role in ubiquitination of CDKN1B/p27kip when associated with CUL4 and SKP2 | 3 | 21.713 | Cleavage |
| 79 | csa-miRn6-3p | Csa7M281360.1 | uncharacterized | XM004150345.1  XM004160200.1 | - | 2 | 5.624 | Cleavage |
| 80 | csa-miRn6-3p | Csa3M000080.1 | kinesin-like calmodulin-binding protein-like | XM004146138.1 | Gene:KCBP; Organism:*Arabidopsis thaliana* ; Minus-end microtubule-dependent motor protein involved in the regulation of cell division and trichome morphogenesis. Possesses basal and microtubule-stimulated ATPase activities.  Interaction of Arabidopsis kinesin-like calmodulin-binding protein with tubulin subunits: modulation by Ca^(2+)^-calmodulin | 3 | 13.868 | Cleavage |
| 81 | csa-miRn6-3p | Csa1M031790.1 | uncharacterized | XM004137522.1 | - | 2 | 13.903 | Cleavage |
| 82 | csa-miRn6-3p | Csa2M116250.1 | putative phospholipid-transporting ATPase 9-like | XM004138878.1 | - | 2.5 | 15.791 | Cleavage |
| 83 | csa-miRn6-3p | Csa6M187950.1 | 50S ribosomal protein L4, chloroplastic-like | XM004160128.1  XM004143099.1 | Protein:50S ribosomal protein L4, chloroplastic;  Gene:RPL4; Organism:*Arabidopsis thaliana* (Mouse-ear cress); Function: This protein binds directly and specifically to 23S rRNA (By similarity). May play a role in plastid transcriptional regulation. | 3 | 6.183 | Cleavage |
| 84 | csa-miRn6-3p | Csa3M636390.1 | uncharacterized | XM004162604.1  XM004144414.1 | - | 2.5 | 16.339 | Cleavage |
| 85 | csa-miRn6-3p | Csa6M382990.1 | uncharacterized | XM004149759.1  XM004168004.1 | - | 2 | 13.342 | Cleavage |
| 86 | csa-miRn6-3p | Csa6M109630.1 | - | - | - | 2 | 2.958 | Cleavage |
| 87 | csa-miRn6-3p | Csa5M648700.1 | ribosomal RNA processing protein 36 homolog | XM004141487.1 | Involved in the early processing steps of the pre-rRNA in the maturation pathway leading to the 18S rRNA. | 2 | 17.573 | Cleavage |
| 88 | csa-miRn6-3p | Csa3M146460.1 | vicilin-like antimicrobial peptides 2-1-like | XM004134389.1 | Protein: Vicilin-like antimicrobial peptides 2-1; Gene: AMP2-1; Organism: *Macadamia integrifolia* (Macadamia nut); Function: Antimicrobial peptides 2b, 2c and 2d have antibacterial and antifungal activity against a range of species. GO - Biological process: defense response to bacterium and fungus; killing of cells of other organism | 3 | 5.406 | Cleavage |
| 89 | csa-miRn6-3p | Csa6M127410.1 | putative DNA repair and recombination protein RAD26-like | XM004139992.1  XM004154548.1 | Protein:DNA repair and recombination protein RAD54-like; Gene:Rad54l; OrganismMus musculus (Mouse); Function: Involved in DNA repair and mitotic recombination. Functions in the recombinational DNA repair (RAD52) pathway. Dissociates RAD51 from nucleoprotein filaments formed on dsDNA. Could be involved in the turnover of RAD51 protein-dsDNA filaments (By similarity). Deficient mice also show significantly shorter telomeres than wild-type controls, indicating that the protein activity plays an essential role in telomere length maintenance in mammals. Deficiency also resulted in an increased frequency of end-to-end chromosome fusions involving telomeres compared to the controls, suggesting a putative role in telomere capping. Non-homologous end joining (NHEJ) and homologous recombination (HR) represent the two major pathways of DNA double-strand break (DSB) repair in eukaryotic cells. LIG4 and RAD54L cooperate to support cellular proliferation, repair spontaneous DSBs, and prevent chromosome and single chromatid aberrations. | 2.5 | 17.445 | Cleavage |
| 90 | csa-miRn6-3p | Csa5M605080.1 | transcription factor HB29-like | XM004135409.1 | - | 2 | 12.312 | Cleavage |
| 91 | csa-miRn6-3p | Csa4M005000.1 | 26.5 kDa heat shock protein, mitochondrial-like | XM004152301.1  XM004171143.1 | Protein:26.5 kDa heat shock protein, mitochondrial;  Gene:HSP26.5; Organism:*Arabidopsis thaliana* (Mouse-ear cress); GO - Biological process: response to heat; response to high light intensity; response to hydrogen peroxide. | 2 | 8.44 | Cleavage |
| 92 | csa-miRn6-3p | Csa7M070780.1 | uncharacterized | XM004159873.1  XM004136883.1 | - | 2.5 | 19.879 | Cleavage |
| 93 | csa-miRn6-3p | Csa6M405900.1 | - | - | - | 2.5 | 0.188 | Cleavage |
| 94 | csa-miRn6-3p | Csa5M180320.1 | uncharacterized | XM004147676.1 | - | 2.5 | 22.425 | Cleavage |
| 95 | csa-miRn6-3p | Csa5M129300.1 | uncharacterized | XM004156033.1  XM004149433.1 | - | 3 | 15.331 | Cleavage |
| 96 | csa-miRn6-3p | Csa2M028470.1 | - | - | - | 2.5 | 6.615 | Cleavage |
| 97 | csa-miRn6-3p | Csa3M740830.1 | uncharacterized | XM004161216.1  XM004137793.1 | - | 2.5 | 4.945 | Cleavage |
| 98 | csa-miRn6-3p | Csa7M024140.1 | uncharacterized | XM004144840.1  XM004153975.1  XM004171274.1 | - | 2.5 | 15.223 | Translation |
| 99 | csa-miRn6-3p | Csa7M341250.1 | uncharacterized | XM004154426.1  XM004150917.1  XM004139660.1 | - | 3 | 16.913 | Translation |
| 100 | csa-miRn6-3p | Csa3M198510.1 | uncharacterized | XM004140764.1  XM004161398.1 | - | 2 | 4.77 | Translation |
| 101 | csa-miRn6-3p | Csa2M073060.1 | LETM1 and EF-hand domain-containing protein 1, mitochondrial-like | XM004155042.1  XM004152789.1 | Human; Crucial for the maintenance of mitochondrial tubular networks and for the assembly of the supercomplexes of the respiratory chain. Required for the maintenance of the tubular shape and cristae organization. | 2.5 | 2.751 | Cleavage |
| 102 | csa-miRn6-3p | Csa7M180130.1 | uncharacterized | XM004170517.1 | - | 2 | 11.755 | Cleavage |
| 103 | csa-miRn6-3p | Csa2M404760.1 | probable metal-nicotianamine transporter YSL7-like | XM004158492.1  XM004138759.1 | May be involved in the transport of nicotianamine-chelated metals. | 3 | 8.907 | Cleavage |
| 104 | csa-miRn6-3p | Csa5M139630.1 | ethylene-responsive transcription factor CRF4-like | XM004145932.1  XM004160356.1 | Protein:AP2-like ethylene-responsive transcription factor SMZ; Gene:SMZ; Organism: *Arabidopsis thaliana* (Mouse-ear cress); Function: Probably acts as a transcriptional activator. Binds to the GCC-box pathogenesis-related promoter element. May be involved in the regulation of gene expression by stress factors and by components of stress signal transduction pathways (By similarity). Repressor of flowering.  Protein: AP2-like ethylene-responsive transcription factor PLT1; Gene: PLT1; Organism: *Arabidopsis thaliana* (Mouse-ear cress); Function: Probably acts as a transcriptional activator. Binds to the GCC-box pathogenesis-related promoter element. May be involved in the regulation of gene expression by stress factors and by components of stress signal transduction pathways (By similarity). Master regulator of basal/root fate. Essential for root quiescent center (QC) and columella specification, stem cell activity, as well as for establishment of the stem cell niche during embryogenesis. Modulates the root polar auxin transport by regulating the distribution of PIN genes. Essential role in respecifying pattern and polarity in damaged roots. Direct target of the transcriptional corepressor TPL. Expression levels and patterns regulated post-transcriptionally by root meristem growth factors (RGFs). | 3 | 12.775 | Cleavage |
| 105 | csa-miRn6-3p | Csa7M388420.1 | pre-mRNA-splicing factor 18-like | XM004146932.1 | Human; Participates in the second step of pre-mRNA splicing. | 3 | 8.666 | Cleavage |
| 106 | csa-miRn6-3p | Csa4M646340.1 | 17.9 kDa class II heat shock protein-like | XM004141286.1  XM004157353.1 | Protein: 17.9 kDa class II heat shock protein;  Gene: HSP17.9-D; Organism: *Glycine max* (Soybean) (Glycine hispida); Biological process: Stress response | 2.5 | 10.411 | Cleavage |
| 107 | csa-miRn6-3p | Csa4M507950.1 | E3 ubiquitin-protein ligase ORTHRUS 2-like | XM004164609.1  XM004146355.1 | Protein: Putative E3 ubiquitin-protein ligase ORTHRUS 4; Gene: ORTH4; Organism: *Arabidopsis thaliana* (Mouse-ear cress); Function: E3 ubiquitin-protein ligase. May participate in CpG methylation-dependent transcriptional regulation | 2.5 | 5.712 | Cleavage |
| 108 | csa-miRn6-3p | Csa6M076750.1 | receptor-like serine/threonine-protein kinase At2g45590-like | XM004145007.1  XM004154251.1  XM004167875.1 | Catalytic activity:  ATP + a protein = ADP + a phosphoprotein. | 2.5 | 6.351 | Translation |
| 109 | csa-miRn6-3p | Csa1M015760.1 | uncharacterized | XM004138288.1 | - | 3 | 17.961 | Cleavage |
| 110 | csa-miRn6-3p | Csa7M425940.1 | putative disease resistance protein RGA4-like | XM004136079.1  XM004169460.1  XM004169461.1 | (Putative disease resistance protein RGA4, *Solanum bulbocastanum* (Wild potato): Disease resistance protein. Resistance proteins guard the plant against pathogens that contain an appropriate avirulence protein via a direct or indirect interaction with this avirulence protein. That triggers a defense system which restricts the pathogen growth.) | 3 | 11.691 | Translation |
| 111 | csa-miRn6-3p | Csa3M144110.1 | proline-rich receptor-like protein kinase PERK8-like | XM004165009.1 | Catalytic activity:  ATP + a protein = ADP + a phosphoprotein. | 2.5 | 5.925 | Cleavage |
| 112 | csa-miRn6-3p | Csa3M104880.1 | uncharacterized | XM004155188.1  XM004133656.1 | - | 3 | 10.246 | Cleavage |
| 113 | csa-miRn6-3p | Csa3M018320.1 | ethylene-responsive transcription factor 6-like | XM004147176.1  XM004164400.1 | - | 3 | 17.199 | Cleavage |
| 114 | csa-miRn6-3p | Csa4M129570.1 | probable receptor-like protein kinase At5g38990-like | XM004154713.1  XM004137211.1 | - | 2.5 | 10.729 | Cleavage |
| 115 | csa-miRn6-3p | Csa7M387720.1 | heat stress transcription factor A-6b-like | XM004146947.1 | LlHSFA1, a novel heat stress transcription factor in lily (*Lilium longiflorum*), can interact with LlHSFA2 and enhance the thermotolerance of transgenic *Arabidopsis thaliana* | 2.5 | 5.968 | Translation |
| 116 | csa-miRn6-3p | Csa3M895940.1 | IWS1-like protein-like | XM004136160.1 | Protein:Protein IWS1 homolog; Gene:Iws1; Organism: *Mus musculus (Mouse);* Function: Transcription factor which plays a key role in defining the composition of the RNA polymerase II (RNAPII) elongation complex and in modulating the production of mature mRNA transcripts. Acts as an assembly factor to recruit various factors to the RNAPII elongation complex and is recruited to the complex via binding to the transcription elongation factor SUPT6H bound to the C-terminal domain (CTD) of the RNAPII subunit RPB1 (POLR2A). The SUPT6H:IWS1:CTD complex recruits mRNA export factors (ALYREF/THOC4, EXOSC10) as well as histone modifying enzymes (such as SETD2) to ensure proper mRNA splicing, efficient mRNA export and elongation-coupled H3K36 methylation, a signature chromatin mark of active transcription | 2.5 | 11.799 | Translation |
| 117 | csa-miRn6-3p | Csa1M073880.1 | L-type lectin-domain containing receptor kinase VII.1-like | XM004146604.1 | - | 2 | 6.08 | Cleavage |
| 118 | csa-miRn6-3p | Csa6M045050.1 | cyanidin-3-O-glucoside 2-O-glucuronosyltransferase-like | XM004140938.1  XM004166754.1 | - | 2 | 7.05 | Translation |
| 119 | csa-miRn6-3p | Csa3M535640.1 | uncharacterized | XM004147966.1 | - | 2 | 8.589 | Cleavage |
| 120 | csa-miRn6-3p | Csa6M324820.1 | - | - | - | 3 | 1.041 | Cleavage |
| 121 | csa-miRn6-3p | Csa6M324820.1 | - | - | - | 3 | 0.075 | Cleavage |
| 122 | csa-miRn6-3p | Csa6M324820.1 | - | - | - | 3 | 0.07 | Cleavage |
| 123 | csa-miRn6-3p | Csa6M324820.1 | - | - | - | 3 | 0.007 | Cleavage |
| 124 | csa-miRn6-3p | Csa6M324820.1 | - | - | - | 3 | 0.005 | Cleavage |
| 125 | csa-miRn6-3p | Csa6M324820.1 | - | - | - | 3 | -1 | Cleavage |
| 126 | csa-miRn6-3p | Csa6M324820.1 | - | - | - | 3 | -1 | Cleavage |
| 127 | csa-miRn6-3p | Csa6M324820.1 | - | - | - | 3 | -1 | Cleavage |
| 128 | csa-miRn6-3p | Csa6M324820.1 | - | - | - | 3 | -1 | Cleavage |
| 129 | csa-miRn6-3p | Csa6M324820.1 | - | - | - | 3 | -1 | Cleavage |
| 130 | csa-miRn6-3p | Csa6M324820.1 | - | - | - | 3 | -1 | Cleavage |
| 131 | csa-miRn6-3p | Csa6M324820.1 | - | - | - | 3 | -1 | Cleavage |
| 132 | csa-miRn6-3p | Csa6M324820.1 | - | - | - | 3 | -1 | Cleavage |
| 133 | csa-miRn6-3p | Csa6M324820.1 | - | - | - | 3 | -1 | Cleavage |
| 134 | csa-miRn6-3p | Csa6M324820.1 | - | - | - | 3 | -1 | Cleavage |
| 135 | csa-miRn6-3p | Csa6M324820.1 | - | - | - | 3 | -1 | Cleavage |
| 136 | csa-miRn6-3p | Csa6M324820.1 | - | - | - | 3 | -1 | Cleavage |
| 137 | csa-miRn6-3p | Csa6M324820.1 | - | - | - | 3 | -1 | Cleavage |
| 138 | csa-miRn6-3p | Csa6M324820.1 | - | - | - | 3 | -1 | Cleavage |
| 139 | csa-miRn6-3p | Csa6M324820.1 | - | - | - | 3 | -1 | Cleavage |
| 140 | csa-miRn6-3p | Csa6M324820.1 | - | - | - | 3 | -1 | Cleavage |
| 141 | csa-miRn6-3p | Csa6M324820.1 | - | - | - | 3 | 0.001 | Cleavage |
| 142 | csa-miRn6-3p | Csa6M324820.1 | - | - | - | 3 | 0.001 | Cleavage |
| 143 | csa-miRn6-3p | Csa6M324820.1 | - | - | - | 3 | 0.002 | Cleavage |
| 144 | csa-miRn6-3p | Csa6M324820.1 | - | - | - | 3 | 0.016 | Cleavage |
| 145 | csa-miRn6-3p | Csa3M646600.1 | uncharacterized | XM004166873.1  XM004144398.1 | - | 2.5 | 13.208 | Cleavage |
| 146 | csa-miRn6-3p | Csa6M079220.1 | ent-kaurenoic acid oxidase 1-like | XM004168024.1  XM004153936.1 | *Arabidopsis thaliana:* Encodes an ent-kaurenoic acid hydroxylase, a member of the CYP88A cytochrome p450 family. | 2.5 | 6.516 | Cleavage |
| 147 | csa-miRn6-3p | Csa3M774740.1 | pleiotropic drug resistance protein 2-like | XM004161196.1 | May be a general defense protein. | 2.5 | 16.527 | Cleavage |
|  | csa-miRn6-3p | Csa3M774740.1 | ABC transporter G family member 34-like | XM004149181.1  XM004137963.1 | - |  |  |  |
| 148 | csa-miRn6-3p | CsaUNM025520.1 | probable long-chain-alcohol O-fatty-acyltransferase 4-like | XM004173323.1  XM004154069.1 | Probable long-chain-alcohol O-fatty-acyltransferase 9 : Catalyzes the final step in the synthesis of long-chain linear esters (waxes). | 2.5 | 4.443 | Cleavage |
| 149 | csa-miRn6-3p | Csa6M181570.1 | UDP-glycosyltransferase 87A1-like | XM004143173.1  XM004169578.1 | - | 2.5 | 10.544 | Translation |
| 150 | csa-miRn6-3p | Csa1M042280.1 | - | - |  | 2 | 0.759 | Cleavage |
| 151 | csa-miRn6-3p | Csa5M650450.1 | F-box/FBD/LRR-repeat protein At1g16930-like | XM004169217.1 | - | 2.5 | 22.584 | Translation |
| 152 | csa-miRn6-3p | Csa7M374620.1 | linoleate 9S-lipoxygenase 1-like | XM004148917.1 | Protein: Linoleate 9S-lipoxygenase 1;  Gene: LOX1.1; Organism: *Hordeum vulgare* (Barley) ; Function: Plant lipoxygenase may be involved in a number of diverse aspects of plant physiology including growth and development, pest resistance, and senescence or responses to wounding. It catalyzes the hydroperoxidation of lipids containing a cis,cis-1,4-pentadiene structure. | 2 | 6.976 | Translation |
|  | csa-miRn6-3p | Csa7M374620.1 | uncharacterized | XM004148898.1 | - |  |  |  |
|  | csa-miRn6-3p | Csa7M374620.1 | uncharacterized, transcript variant 3 | XR181007.1 | - |  |  |  |
|  |  |  | uncharacterized, transcript variant 1 | XR181005.1 |  |  |  |  |
| 153 | csa-miRn6-3p | Csa7M394700.1 | uncharacterized, misc RNA | XR180752.1  XR180993.1 | - | 3 | 15.076 | Cleavage |
| 154 | csa-miRn6-3p | Csa5M156130.1 | uncharacterized | XM004159933.1  XM004143948.1 | - | 3 | 10.727 | Cleavage |
| 155 | csa-miRn6-3p | Csa6M301610.1 | - | - | - | 2.5 | 10.351 | Translation |
| 156 | csa-miRn6-3p | Csa7M037490.1 | - | - | - | 3 | 19.835 | Cleavage |
| 157 | csa-miRn6-3p | Csa1M467730.1 | - | - | - | 2.5 | 10.037 | Cleavage |
| 158 | csa-miRn6-3p | Csa5M601600.1 | uncharacterized | XM004164346.1  XM004135193.1 | - | 3 | 6.503 | Cleavage |
| 159 | csa-miRn6-3p | Csa5M605000.1 | transcription factor TCP14-like | XM004135413.1 | Transcription factor AtTCP14 regulates embryonic growth potential during seed germination in *Arabidopsis thaliana*.  Analysis of functional redundancies within the Arabidopsis TCP transcription factor family | 2 | 8.996 | Translation |
|  |  |  | transcription factor TCP15-like | XM004173579.1 |  |  |  |  |
| 160 | csa-miRn6-3p | Csa7M206930.1 | uncharacterized | XM004169859.1  XM004139814.1 | - | 3 | 23.886 | Cleavage |
| 161 | csa-miRn6-3p | Csa5M640550.1 | receptor-like protein kinase FERONIA-like | XM004141542.1  XM004156169.1 | *Arabidopsis thaliana*: Receptor-like protein kinase that mediates the female control of male gamete delivery during fertilization, including growth cessation of compatible pollen tubes ensuring a reproductive isolation barriers, by regulating MLO7 subcellular polarization upon pollen tube perception in the female gametophyte synergids. Required for cell elongation during vegetative growth, mostly in a brassinosteroids- (BR-) independent manner. Acts as an upstream regulator for the Rac/Rop-signaling pathway that controls ROS-mediated root hair development. Seems to regulates a cross-talk between brassinosteroids and ethylene signaling pathways during hypocotyl elongation. Negative regulator of brassinosteroid response in light-grown hypocotyls, but required for brassinosteroid response in etiolated seedlings. Mediates sensitivity to powdery mildew (e.g. *Golovinomyces orontii*). Positive regulator of auxin-promoted growth that represses the abscisic acid (ABA) signaling via the activation of ABI2 phosphatase. Required for RALF1-mediated extracellular alkalinization in a signaling pathway preventing cell expansion. | 3 | 7.359 | Translation |
| 162 | csa-miRn6-3p | Csa6M106750.1 | uncharacterized | XM004140459.1  XM004165916.1 | - | 3 | 14.357 | Cleavage |
| 163 | csa-miRn6-3p | Csa1M600110.1 | 17.6 kDa class I heat shock protein-like | XM004159294.1  XM004144095.1 | 17.6 kDa class Ⅱheat shock protein: response to heat | 2.5 | 0.617 | Cleavage |
|  | csa-miRn6-3p | Csa1M600110.1 | uncharacterized | XM004144094.1 | - |  |  |  |
| 164 | csa-miRn6-3p | Csa2M361860.1 | uncharacterized | XM004152724.1 | - | 2.5 | 14.565 | Translation |
| 165 | csa-miRn6-3p | Csa1M002840.1 | uncharacterized | XM004138214.1 | - | 2.5 | 9.878 | Translation |
| 166 | csa-miRn6-3p | Csa1M524700.1 | uncharacterized | XM004145282.1  XM004163761.1  XM004153101.1 | - | 3 | 17.979 | Cleavage |
| 167 | csa-miRn6-3p | Csa7M133420.1 | - | - | - | 2 | 7.226 | Translation |
| 168 | csa-miRn6-3p | Csa6M088010.1 | uncharacterized | XM004140528.1 | - | 3 | 21.927 | Cleavage |
| 169 | csa-miRn6-3p | Csa3M131930.1 | 60S ribosomal protein L13a-2-like | XM004169463.1  XM004133947.1 | *Arabidopsis thaliana* ; GO - Molecular function:  structural constituent of ribosome | 3 | 14.127 | Cleavage |
| 170 | csa-miRn6-3p | Csa1M006330.1 | uncharacterized | XM004154951.1 |  | 2.5 | 20.52 | Cleavage |
| 171 | csa-miRn6-3p | Csa3M901080.1 | ABC transporter B family member 2-like | XM004136350.1  XM004165274.1 | *Arabidopsis thaliana:* ABC transporter B family member 1 : Auxin efflux transporter that acts as a negative regulator of light signaling to promote hypocotyl elongation. Mediates the accumulation of chlorophyll and anthocyanin, as well as the expression of genes in response to light. Participates directly in auxin efflux and thus regulates the polar (presumably basipetal) auxin transport (from root tips to root elongating zone). Transports also some auxin metabolites such as oxindoleacetic acid and indoleacetaldehyde. Involved in divers auxin-mediated responses including gravitropism, phototropism and lateral root formation. Confers resistance to herbicides such as dicamba, pendimethalin, oryzalin, and monosodium acid methanearsonate (MSMA), but not to herbicides such as glyphosate, atrazine, bentazon and fluazifop-p-butyl. Mediates also resistance to xenobiotics such as cycloheximide and the cytokinin N6-(2-isopentenyl)adenine (2IP). | 2.5 | 1.304 | Translation |
| 172 | csa-miRn6-3p | Csa1M015600.1 | uncharacterized | XM004138281.1 | - | 2.5 | 12.203 | Translation |
| 173 | csa-miRn6-3p | Csa6M205820.1 | insulin-degrading enzyme-like | XM004165688.1  XM004150557.1 | Insulin-degrading enzyme (IDE): A novel heat shock-like protein: Insulin-Degrading Enzyme (IDE) is a highly conserved zinc metallopeptidase which is ubiquitously distributed in human tissues, being particularly abundant in the brain, liver and muscles. IDE activity has been historically associated to insulin and β-amyloid catabolism. However, over the last decade, several experimental findings have established that IDE is also involved in a wide variety of physiopathological processes, including ubiquitin clearance and Varicella Zoster Virus infection. In this study, we demonstrate that normal and malignant cells exposed to different stresses markedly up-regulate IDE in a Heat Shock Protein (HSP)-like fashion. Additionally, we focused our attention on tumor cells and report that (i) IDE is overexpressed in vivo in tumors of the Central Nervous System (CNS); (ii) IDE-silencing inhibits neuroblastoma (SHSY5Y) cell prolifera- tion and triggers cell death; (iii) IDE inhibition is accompanied by a decrease of the poly-ubiquitinated protein content and co-immunoprecipitates with proteasome and ubiquitin in SHSY5Y cells. In this work, we propose a novel role for IDE as a Heat-Shock Protein with implications in cell growth regulation and cancer progression, thus opening up an intriguing hypothesis of IDE as an anticancer target. | 2.5 | 13.16 | Cleavage |
| 174 | csa-miRn6-3p | Csa6M091910.1 | RNA polymerase II C-terminal domain phosphatase-like 3-like | XM004140603.1  XM004157585.1 | Protein: RNA polymerase II C-terminal domain phosphatase-like 1; Gene: CPL1; Organism: *Arabidopsis thaliana* (Mouse-ear cress); Function: Processively dephosphorylates 'Ser-5' but not 'Ser-2' of the heptad repeats YSPTSPS in the C-terminal domain of the largest RNA polymerase II subunit (RPB1). This promotes the activity of RNA polymerase II. Together with CPL2, required for male gametes fertility. Multifunctional regulator that modulates plant growth, stress, and phytohormones responses. Negative regulator of stress gene transcription involved in abscisic acid (ABA) mediated and jasmonic acid (JA) mediated signaling pathways, NaCl, osmotic stress, wounding, and cold resistance. Regulates negatively the expression of jasmonic acid (JA) biosynthetic genes in response to wounding.  Protein: RNA polymerase II C-terminal domain phosphatase-like 4; Gene: CPL4; Organism: *Arabidopsis thaliana* (Mouse-ear cress); Function: Processively dephosphorylates 'Ser-2' and/or 'Ser-5' of the heptad repeats YSPTSPS in the C-terminal domain of the largest RNA polymerase II subunit (RPB1). This promotes the activity of RNA polymerase II (By similarity). Required for normal plant growth.  A novel RNA polymerase IIC-terminal domain phosphatase that preferentially dephosphorylates serine 5 | 3 | 15.995 | Cleavage |
| 175 | csa-miRn6-3p | Csa5M601600.2 | uncharacterized | XM004164346.1  XM004135193.1 | - | 3 | 6.503 | Cleavage |
| 176 | csa-miRn6-3p | Csa7M339700.1 | - | - | - | 3 | 7.557 | Cleavage |
| 177 | csa-miRn6-3p | Csa3M002870.1 | - | - | - | 2 | 4.052 | Cleavage |
| 178 | csa-miRn6-3p | Csa4M022990.1 | methyltransferase-like protein 1-like | XM004146892.1  XM004161549.1  XM004167710.1 | methyltransferase activity | 3 | 14.084 | Cleavage |
| 179 | csa-miRn6-3p | Csa4M045040.1 | probable dehydrin LEA-like | XM004169589.1 | Organism: *Arabidopsis thaliana*; GO-Biological process: response to stress and water; | 3 | 18.078 | Cleavage |
|  | csa-miRn6-3p | Csa4M045040.1 | dehydrin Rab 18-like | XM004146501.1 | Structure and organization of two closely related low-temperature-induced dhn/lea/rab-like genes in *Arabidopsis thaliana* L. Heynh  Accumulation of dehydrin-like proteins in the mitochondria of cereals in response to cold, freezing, drought and ABA treatment. |  |  |  |
| 180 | csa-miRn6-3p | Csa6M133770.1 | ethylene-responsive transcription factor CRF4-like | XM004154508.1  XM004139978.1 | Protein:AP2-like ethylene-responsive transcription factor SMZ; Gene:SMZ; Organism: *Arabidopsis thaliana* (Mouse-ear cress); Function: Probably acts as a transcriptional activator. Binds to the GCC-box pathogenesis-related promoter element. May be involved in the regulation of gene expression by stress factors and by components of stress signal transduction pathways (By similarity). Repressor of flowering.  Protein: AP2-like ethylene-responsive transcription factor PLT1; Gene: PLT1; Organism: *Arabidopsis thaliana* (Mouse-ear cress); Function: Probably acts as a transcriptional activator. Binds to the GCC-box pathogenesis-related promoter element. May be involved in the regulation of gene expression by stress factors and by components of stress signal transduction pathways (By similarity). Master regulator of basal/root fate. Essential for root quiescent center (QC) and columella specification, stem cell activity, as well as for establishment of the stem cell niche during embryogenesis. Modulates the root polar auxin transport by regulating the distribution of PIN genes. Essential role in respecifying pattern and polarity in damaged roots. Direct target of the transcriptional corepressor TPL. Expression levels and patterns regulated post-transcriptionally by root meristem growth factors (RGFs). | 2.5 | 18.722 | Cleavage |
| 181 | csa-miRn6-3p | Csa1M025140.3 | DEAD-box ATP-dependent RNA helicase 42-like transcript variant 1 | XM004137301.1 | DEAD-box ATP-dependent RNA helicase 39 [ *Arabidopsis thaliana* (thale cress) ]: Encodes RH39, a DEAD-box protein involved in the introduction of the hidden break into the 23S rRNA in the chloroplasts. Recombinant RH39 binds to the 23S rRNA in a segment adjacent to the stem-loop creating the hidden break target loop in a sequence-dependent manner. Has ATP-hydrolyzing activity at a Kcat of 5.3 /min in the presence of rRNA sequence. Mutants have drastically reduced level of level of ribulose 1,5-bisphosphate carboxylase/oxygenase.  Pisum sativum p68 DEAD-box protein is ATP-dependent RNA helicase and unique bipolar DNA helicase | 3 | 2.832 | Translation |
| 182 | csa-miRn6-3p | Csa1M025140.2 | DEAD-box ATP-dependent RNA helicase 42-like transcript variant 1 | XM004137301.1 |  | 3 | 2.832 | Translation |
| 183 | csa-miRn6-3p | Csa1M025140.1 | DEAD-box ATP-dependent RNA helicase 42-like transcript variant 1 | XM004137301.1 |  | 3 | 2.832 | Translation |
| 184 | csa-miRn6-3p | Csa1M025140.4 | DEAD-box ATP-dependent RNA helicase 42-like transcript variant 1 | XM004137301.1 |  | 3 | 2.832 | Translation |
| 185 | csa-miRn6-3p | Csa6M483370.2 | phosphatidylinositol 4-kinase beta 1-like | XM004148606.1  XM004163003.1 | Gene: PI4KB; Organism: *Homo sapiens* (Human); Function: Phosphorylates phosphatidylinositol (PI) in the first committed step in the production of the second messenger inositol-1,4,5,-trisphosphate (PIP). May regulate Golgi disintegration/reorganization during mitosis, possibly via its phosphorylation. Involved in Golgi-to-plasma membrane trafficking (By similarity).  Class III phosphatidylinositol 4-kinase alpha and beta are novel host factor regulators of hepatitis C virus replication. | 3 | 16.221 | Cleavage |
| 186 | csa-miRn6-3p | Csa6M483370.1 | phosphatidylinositol 4-kinase beta 1-like | XM004148606.1  XM004163003.1 |  | 3 | 16.221 | Cleavage |
| 187 | csa-miRn6-3p | Csa6M133770.2 | ethylene-responsive transcription factor CRF4-like | XM004154508.1  XM004139978.1 | Protein:AP2-like ethylene-responsive transcription factor SMZ; Gene:SMZ; Organism: *Arabidopsis thaliana* (Mouse-ear cress); Function: Probably acts as a transcriptional activator. Binds to the GCC-box pathogenesis-related promoter element. May be involved in the regulation of gene expression by stress factors and by components of stress signal transduction pathways (By similarity). Repressor of flowering.  Protein: AP2-like ethylene-responsive transcription factor PLT1; Gene: PLT1; Organism: *Arabidopsis thaliana* (Mouse-ear cress); Function: Probably acts as a transcriptional activator. Binds to the GCC-box pathogenesis-related promoter element. May be involved in the regulation of gene expression by stress factors and by components of stress signal transduction pathways (By similarity). Master regulator of basal/root fate. Essential for root quiescent center (QC) and columella specification, stem cell activity, as well as for establishment of the stem cell niche during embryogenesis. Modulates the root polar auxin transport by regulating the distribution of PIN genes. Essential role in respecifying pattern and polarity in damaged roots. Direct target of the transcriptional corepressor TPL. Expression levels and patterns regulated post-transcriptionally by root meristem growth factors (RGFs). | 2.5 | 18.722 | Cleavage |
| 188 | csa-miRn6-3p | Csa5M139210.1 | dof zinc finger protein DOF4.6-like | XM004174050.1  XM004145894.1 | Gene: DOF4.6; Organism: *Arabidopsis thaliana* (Mouse-ear cress); Function: Transcription factor that binds specifically to a 5'-AA[AG]G-3' consensus core sequence. | 3 | 11.023 | Cleavage |
| 189 | csa-miRn6-3p | Csa7M433320.1 | uncharacterized | XM004136031.1 | - | 2.5 | 20.2 | Translation |
| 190 | csa-miRn6-3p | Csa3M829150.1 | chloroplastic group IIA intron splicing facilitator CRS1, chloroplastic-like | XM004147432.1  XM004164607.1 | Gene: CRS1; Organism: *Zea mays* (Maize); Function: Required for the splicing of group IIA introns in chloroplasts, and especially for atpF, by regulating the intron folding. Forms splicing particles with RNA. Also involved in chloroplast protein translation. | 3 | 9.513 | Cleavage |
| 191 | csa-miRn6-3p | Csa7M448690.1 | uncharacterized | XM004141675.1  XM004170674.1  XM004155913.1 | - | 3 | 23.504 | Cleavage |
| 192 | csa-miRn6-3p | Csa4M001840.1 | U1 small nuclear ribonucleoprotein A-like | XM004156787.1  XM004152198.1 | Autoimmune response to U1 small nuclear ribonucleoprotein (U1 snRNP) associated with cytomegalovirus infection. | 2.5 | 18.668 | Cleavage |
| 193 | csa-miRn6-3p | Csa1M136770.1 | - | - | - | 2.5 | 6.382 | Cleavage |
| 194 | csa-miRn6-3p | Csa7M270590.4 | uncharacterized | XM004150979.1  XM004168065.1  XM004172656.1 | - | 3 | 15.389 | Translation |
| 195 | csa-miRn6-3p | Csa7M270590.2 | uncharacterized | XM004150979.1  XM004168065.1  XM004172656.1 | - | 3 | 15.389 | Translation |
| 196 | csa-miRn6-3p | Csa7M270590.1 | uncharacterized | XM004150979.1  XM004168065.1  XM004172656.1 | - | 3 | 15.389 | Translation |
| 197 | csa-miRn6-3p | Csa7M270590.3 | uncharacterized | XM004150979.1  XM004168065.1  XM004172656.1 | - | 3 | 15.389 | Translation |
| 198 | csa-miRn6-3p | Csa5M647440.1 | dynamin-2B-like | XM004156145.1  XM004141479.1 | Protein：Dynamin-2B; Gene：DRP2B; Organism: *Arabidopsis thaliana* (Mouse-ear cress); Function: Protein：ABSCISIC ACID-INSENSITIVE 5-like protein 2; Gene：DPBF3; Organism: *Arabidopsis thaliana* (Mouse-ear cress); Function: Putative microtubule-associated force-producing protein, able to bind and hydrolyze GTP. | 2.5 | 9.652 | Cleavage |
| 199 | csa-miRn6-3p | Csa1M136780.1 | uncharacterized | XM004139515.1 | - | 2 | 16.174 | Translation |
| 200 | csa-miRn6-3p | Csa1M599530.1 | uncharacterized | XM004144024.1  XM004159301.1 | - | 2 | 16.73 | Translation |
| 201 | csa-miRn6-3p | Csa3M149380.1 | - | - | - | 3 | 2.645 | Translation |
| 202 | csa-miRn6-3p | Csa4M377720.1 | protein ABSCISIC ACID-INSENSITIVE 5-like | XM004142636.1  XM004163630.1 | Protein：ABSCISIC ACID-INSENSITIVE 5-like protein 2; Gene：DPBF3; Organism: *Arabidopsis thaliana* (Mouse-ear cress); Function: Binds to the embryo specification element and the ABA-responsive element (ABRE) of the Dc3 gene promoter. Could participate in abscisic acid-regulated gene expression during seed development. | 2.5 | 19.118 | Cleavage |
| 203 | csa-miRn6-3p | Csa6M511820.1 | uncharacterized | XM004134740.1  XM004155570.1 | - | 2.5 | 5.04 | Cleavage |
| 204 | csa-miRn6-3p | Csa1M398140.1 | tudor domain-containing protein 3-like | XM004173146.1 | - | 3 | 12.239 | Cleavage |
| 205 | csa-miRn6-3p | Csa3M036440.1 | - | - | - | 3 | 4.951 | Cleavage |
| 206 | csa-miRn6-3p | Csa6M128630.1 | ELMO domain-containing protein A-like | XM004154554.1  XM004139888.1 | - | 3 | 1.926 | Cleavage |
| 207 | csa-miRn8-3p | Csa3M849900.1 | glutamate receptor 2.7-like | XM004154825.1  XM004146807.1 | A Rice Glutamate Receptor–Like Gene Is Critical for the Division and Survival of Individual Cells in the Root Apical Meristem | 2.5 | 14.171 | Cleavage |
| 208 | csa-miRn8-3p | Csa3M806260.1 | probable L-type lectin-domain containing receptor kinase S.5-like | XM004136685.1 | - | 3 | 11.745 | Cleavage |
| 209 | csa-miRn8-3p | Csa7M257340.1 | polyol transporter 5-like | XM004148254.1  XM004159107.1 | Arabidopsis POLYOL TRANSPORTER5, a new member of the monosaccharide transporter-like superfamily, mediates H+-Symport of numerous substrates, including myo-inositol, glycerol, and ribose. | 3 | 8.499 | Cleavage |
| 210 | csa-miRn8-3p | Csa7M432440.1 | pre-mRNA-processing-splicing factor 8-like | XM004135796.1  XM004158735.1 | Functions as a scaffold that mediates the ordered assembly of spliceosomal proteins and snRNAs. Required for the assembly of the U4/U6-U5 tri-snRNP complex. Functions as scaffold that positions spliceosomal U2, U5 and U6 snRNAs at splice sites on pre-mRNA substrates, so that splicing can occur. Interacts with both the 5' and the 3' splice site. | 2.5 | 15.058 | Translation |
| 211 | csa-miRn8-3p | Csa5M161290.1 | nitrate transporter 1.1-like | XM004147569.1 | Nitrate signalling mediated by the NRT1.1 nitrate transporter antagonises l-glutamate-induced changes in root architecture | 3 | 9.58 | Cleavage |

Note: “-”, function annotation is unknown.
