# Supplementary material for: Expression profiling and regulatory network of cucumber microRNAs and their putative target genes in response to cucumber green mottle mosaic virus infection
Source: Arch Virol. 2019 Feb 24;164(4):1121–34. doi: 10.1007/s00705-019-04152-w (PMC6420491; doi:10.1007/s00705-019-04152-w)
Supplement: Supplementary file 5 — Supplementary material 5 (DOCX 931 kb) [file 705_2019_4152_MOESM5_ESM.docx]

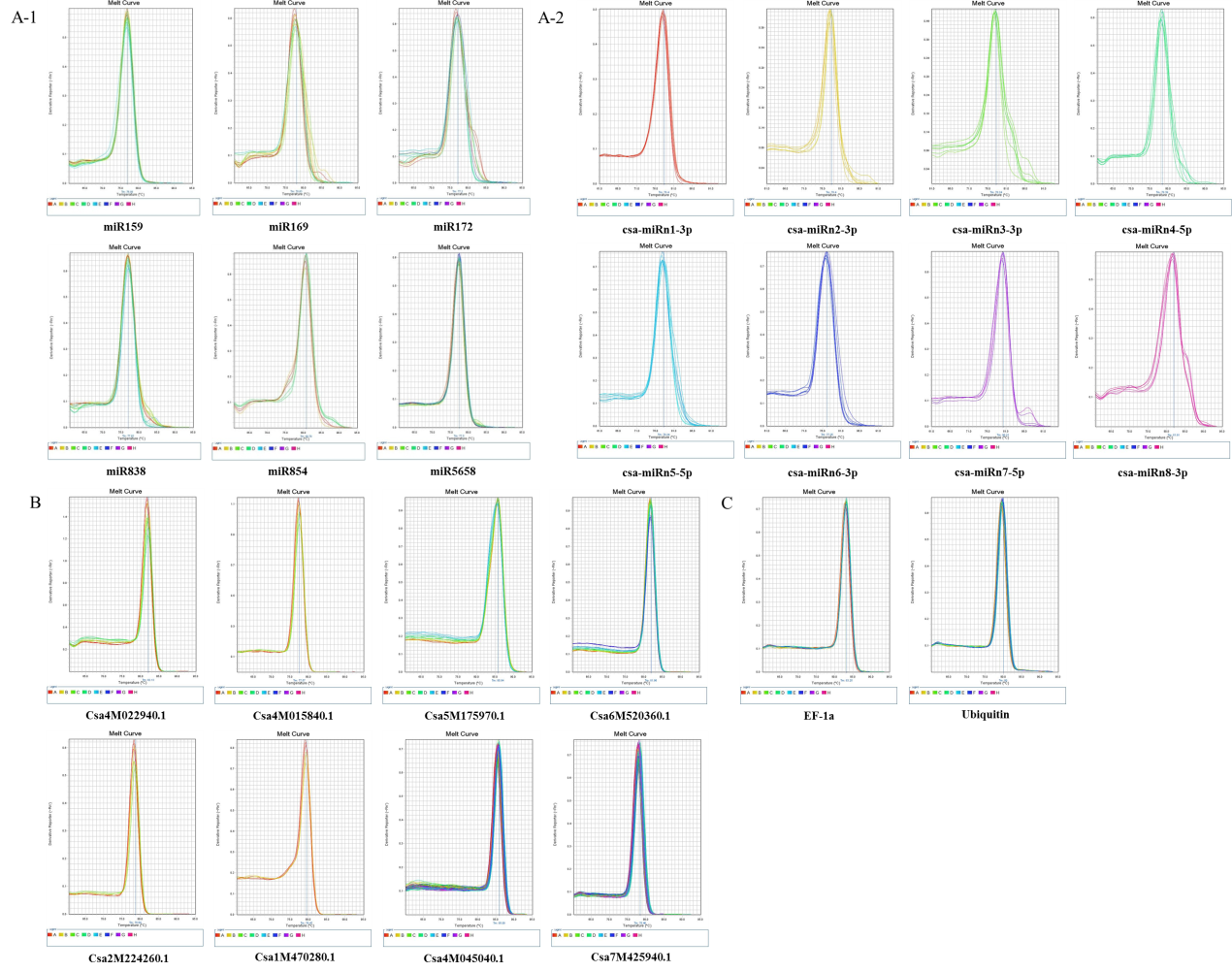
Supplementary material 5 Fig. S1 Melting curves of miRNAs (A-1, known miRNAs; A-2, novel miRNAs), target genes (B) and reference genes (C).
