# Supplementary material for: Expression profiling and regulatory network of cucumber microRNAs and their putative target genes in response to cucumber green mottle mosaic virus infection
Source: Arch Virol. 2019 Feb 24;164(4):1121–34. doi: 10.1007/s00705-019-04152-w (PMC6420491; doi:10.1007/s00705-019-04152-w)
Supplement: Supplementary file 6 — Supplementary material 6 (DOCX 294 kb) [file 705_2019_4152_MOESM6_ESM.docx]

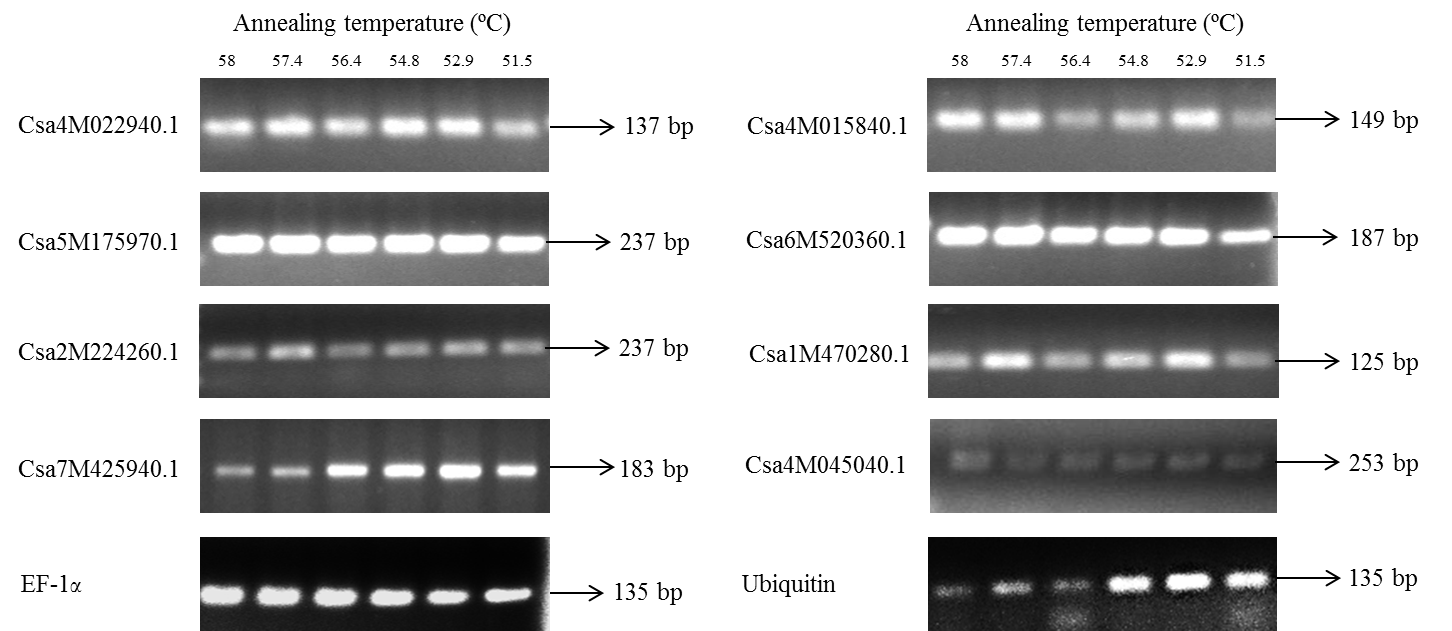
Supplementary material 6 Fig. S2 Agarose gel electrophoresis of PCR products of eight target genes and two reference genes with gradient annealing temperatures (from 58ºC to 52ºC) by RT-PCR.
